# Study on the activation of cell death mechanisms: in search of new therapeutic targets in glioblastoma multiforme

**DOI:** 10.1007/s10495-023-01857-x

**Published:** 2023-05-27

**Authors:** Ludovica Gaiaschi, Cristina Favaron, Claudio Casali, Federica Gola, Fabrizio De Luca, Mauro Ravera, Elisa Roda, Paola Rossi, Maria Grazia Bottone

**Affiliations:** 1 Laboratory of Cell Biology and Neurobiology, Department of Biology and Biotechnology “L. Spallanzani”, University of Pavia, Via Ferrata 9, 27100 Pavia, Italy; 2 Department of Sciences and Technological Innovation (DiSIT), University of Piemonte Orientale “A. Avogadro”, Viale Teresa Michel 11, 15121 Alessandria, Italy; 3 Laboratory of Clinical and Experimental Toxicology, Pavia Poison Centre, National Toxicology Information Centre, Toxicology Unit, ICS Maugeri Spa, IRCCS Pavia, Via Maugeri 10, Pavia, Italy; 4 Laboratory of Neurophysiology and Integrated Physiology, Department of Biology and Biotechnology “L. Spallanzani”, University of Pavia, Via Ferrata 9, 27100 Pavia, Italy

**Keywords:** Glioblastoma Multiforme, Phytotherapy, Combined therapy, Cell death pathway

## Abstract

Malignant primary brain tumors remain among the most difficult cancers to treat, in particular, Glioblastoma Multiforme (GBM) is the deadliest brain tumor. The standard therapies currently used are not efficient enough in improving patients’ survival and quality of life. Cisplatin (CDDP), a platinum-based drug, has shown efficacy against different solid neoplasms, but it is also associated to different forms of off-target toxicity. To overcome the limitation in the use of CDDP in the treatment of GBM patients, fourth generation platinum compounds are been synthesized, one of them is the Pt(IV)Ac-POA, a prodrug with a medium-chain fatty acid as axial ligand, which acts as a histone 3 deacetylase inhibitor. Moreover, recently, the antioxidant effects of medicinal mushrooms have been shown to induce a lowering of the toxicity of chemotherapy drugs, inducing greater therapeutic efficiency, thus the combined therapy of chemotherapy and micotherapy could be helpful in the treatment of GBM reducing the adverse effects of the former thanks to phytotherapy’s antioxidant, anti-inflammatory, immunomodulatory and antitumoral activities. Here, through immunoblotting, ultrastructural and immunofluorescence analysis, we evaluated the contribution in the activation of different cell death pathway of Micotherapy U-Care, a medicinal blend supplement, used together with platinum-based compounds on human glioblastoma U251 cells.

**Supplementary Information:**

The online version contains supplementary material available at 10.1007/s10495-023-01857-x.

## Introduction

Malignant primary brain tumors remain among the most difficult cancers to treat, with an overall survival to 5 years by no more than 35%. The most common malignant primary brain tumors in adults are gliomas, Glioblastoma Multiforme (GBM) belongs to this category. GBM is the deadliest brain tumor, with a 5-year mortality greater than 90% and with a mean survival of 12.6 months [1]. It is, according to Classification of Central Nervous System tumors updated in 2016 by the World Health Organization, a grade IV astrocytoma [2]. 

The standard therapeutic approach to GBM care includes surgical resection, radiotherapy and chemotherapy. Obtain a radical destruction of the cancer mass is very difficult due to both the invasive nature of the cancer cells and because GBMs are often located in eloquent areas of the brain. Temozolomide (TMZ) is an alkylating agent, used for chemotherapy. The mechanism action involves the addition of a methyl group to purine and pyrimidine of DNA, inducing cell damage and death. However, many patients with GBM resist to the treatment with Temozolomide via O-6-methylguanine-DNA methyltransferase (MGMT), a DNA repair protein [3]. Platinum-based drugs, such as cisplatin (cis-diaminedicloroplatinum-II, CDDP), that has shown efficacy against different solid neoplasms, are also among the chemotherapy agents used the most. Although cisplatin exerts a powerful antineoplastic activity it is also associated to different forms of off-target toxicity such as: nephrotoxicity, neurotoxicity and ototoxicity; but other disorders can also be triggered such as: myelosuppression, nausea and emesis [4].

To overcome the limitation in the use of CDDP, other fourth generation platinum compounds are synthesized. They maintain the equatorial coordination sphere Pt (II) and present ligands in an axial position, designed to enhance the pharmacological properties of the complex. Among these compounds, there is (OC-6-44)-acetatodiamineediclorido (2-(2-propinyl)octanoate) platinum (IV), known as Pt(IV)Ac-POA. The prodrug contains as axial ligands a medium-chain fatty acid, which acts as a deacetylase inhibitor (POA) and an inert acetate (Ac). It has been established that POA, due to its action as histone deacetylase (HDAC) inhibitor, is an inducer of histone H3 a hyperacetylation on lysine 9, helping to increase the overall antiproliferative activity [5].

A new possibility to improve GBM patients’ survival and collateral effects’ reduction can be represented by combined treatments, as new platinum-based compounds and phytotherapy. Micotherapy is a branch of herbal medicine that involves the use of medicinal mushrooms, so far some mushrooms are valued for their pharmacological properties, such as: antimicrobial, antioxidant, anti-inflammatory, immunomodulatory, cytotoxic, hepatoprotective and antitumoral [6].

In this paper we want to evaluate the contribution in the activation of different cell death pathway of Micotherapy U-Care, a medicinal blend supplement, consisting of several extracts of mycelium and sporophores of Agaricus blazei, Cordyceps sinensis, Grifola frondose, Lentinula edodes and Ganoderma lucidum [7].

Cell death is a physiological process that plays a key role in development, remodeling, maintenance of homeostasis, tissue functionality and defense mechanisms. Cell death mechanisms can be classified into two main groups: programmed pathway and not controlled ones [8]. The study of these pathways is essential for the enrichment of knowledge related to different pathologies and for the development of molecules of pharmaceutical interest.

## Material and methods

### Cell culture and treatment for U251 MG cell line

Human glioblastoma U251 MG cells were cultured in Eagle’s minimal essential medium supplemented with fetal bovine serum, glutamine, sodium pyruvate, non-essential amino acids solution, penicillin and streptomycin in a 5% CO_2_ humidified atmosphere at 37 °C, as previously described. Cells were plated as 4*10^6^ cells in T75 flasks, or 6*10^5^ cells on coverslips positioned in 6-well plates and treated after 48 h having reached the 80% of confluence. The cultured cells were treated for 48 h with CDDP 40 μM or Pt(IV)Ac-POA 10 μM alone or in combination with Micotherapy U-Care 5 mg/ml at continuous exposure; usage concentrations were chosen considering previous experimental data [9–11].

### Cell viability assay: trypan blue-exclusion method

U251 cells were plated at density of 6*10^5^ cells/well in 6 wells plate and treated as already indicated; after 48 h of continuous exposure at the different therapies the cells were incubated with clean completed medium for 7 days (recovery condition). Cell viability was evaluated at 3 different time points: after 2 h of exposure to the therapies (t1), after 48 h of treatment (t2), and after the end of the recovery period (t3). For this assay, the capability of a vital dye, such as Trypan blue, to cross compromise membranes was exploited. In particular, cells were collected after mild trypsinization and centrifuged at 600 rpm for 10 min. The pellet was resuspended in 1 ml of PBS and 5 μl of suspension was mixed with 5 μl of 0.2% Trypan blue to check the cell viability. The cells’ count was effectuated with Burker counting chamber.

### Scratch wound healing migration assay

Cell migration was assessed using a scratch wound healing assay. After 48 h of continuous treatment cells in adhesion were collected by mild trypsinization and counted. 6*10^5^ cells were cultured to a monolayer in six-well plates for 12 h and wounded using sterile 10 μl pipette tips. Cells were washed with PBS to remove any debris. Photos were captured at 0 (t0), 6 (t2), 12 (t3), and 24 (t4) h after wounding. The gap distance was evaluated using ImageJ software. The area of each time was normalized to the t0, the percentage of wound closure in the time was analyzed.

### Immunofluorescence reactions

Cells grown on coverslips, after treatments, were fixed with 4% formaldehyde AT and with 70% ethanol at − 20 °C. Samples were prepared and subjected to immunofluorescence-staining according to the protocol described in previous works [11]. The used primary antibodies (Table 1) were prepared in PBS-Tween 0.2% and revealed with solution of the secondary antibodies (Alexa 594 or 488 conjugated anti-mouse or anti-rabbit antibody, Alexa Fluor, Molecular Probes, Invitrogen) diluted at 1:200 in PBS-Tween 0.2%. Nuclei were counterstained with 0.1 μg/ml of Hoechst 33,258 and coverslips were mounted with Mowiol (Calbiochem).

**Table 1:** Primary antibodies used for fluorescence immunocytochemistry reactions (ICC) and western blot reactions (WB)

Antigen	Primary antibody	Dilution
AIF	Rabbit polyclonal anti-AIF (Cell Signaling Technology, Danvers, USA)	ICC 1:200
Caspase8	Mouse monoclonal anti-Casp8 (Cell Signaling Technology, Danvers, USA)	ICC 1:100 WB 1:500
Caspase9	Rabbit monoclonal anti-Casp8 (Cell Signaling Technology, Danvers, USA)	WB 1:500
Caspase3	Mouse monoclonal anti-Casp3 (Cell Signaling Technology, Danvers, USA)	ICC 1:100 WB 1:1000
PARP1	Rabbit monoclonal anti-PARP1 (Cell Signaling Technology, Danvers, USA)	ICC 1:200
LC3B	Rabbit polyclonal anti-LC3B (Cell Signaling Technology, Danvers, USA)	ICC 1:400 WB 1:1500
SQSTM1/p62	Mouse monoclonal anti-SQQTM1/p62 (Abcam, Cambridge, UK)	ICC 1:100 WB 1:1000
PINK1	Rabbit polyclonal anti-PINK1 (Abcam, Cambridge, UK)	ICC 1:500
Parkin	Rabbit polyclonal anti-Parkin (Abcam, Cambridge, UK)	ICC 1:500
RIP1	Rabbit polyclonal anti-RIP1 (Santa Cruz Biotechnology, Dallas, USA)	ICC 1:500
MLKL	Rat monoclonal anti-MLKL clone 3H1 (Merck Millipore, Burlington, USA)	ICC 1:200
Mitochondria	Human autoimmune serum recognizing the 70 kDa E2 subunit of pyruvate dehydrogenase complex	ICC 1:200
Lysosomes	Human autoimmune serum recognizing lysosomal proteinase	ICC 1:400
Alpha-tubulin	Mouse monoclonal anti-α-tubulin (Cell Signaling Technology, Danvers, USA)	ICC 1:1000
Beta-actin	Rabbit polyclonal anti-β-actin (GeneTex, Irvine, USA)	ICC 1:300 WB 1:2000

### Fluorescence microscopy and fluorescence intensity determination

An Olympus BX51 microscope was used. Images were recorded with an Olympus MagnaFire camera system and processed with the Olympus Cell F software. ImageJ Program was used to measure fluorescence intensity.

### Western blot analyses

Treated and control cells were washed twice with PBS and lysed in a buffer containing Tris HCl 1 M pH 7.6, MgCl_2_ 1 M, NP40 Nonidet 100%, DTT 1 M and β-Glycerophosphate 1 M with the addition of proteases and phosphatases inhibitors at 4 °C for 8 min. Proteins were quantified using the Bradford reagent (Sigma Aldrich, Italy). After that, an appropriate amount of mix, constituted by Tris-HCl 1 M, SDS 10X, Glycerol, DTT 1 M and Bromophenol blue, was added to the samples. 45 μg of samples were electrophoresed in 10% or 12% SDS-PAGE mini-gels according to the molecular weight of the protein of interest and transferred onto a nitrocellulose membrane (BioRad, Hercules, CA) by semidry blotting for 1.30 h under constant current. The membranes were saturated for 30 min with PBS containing 0.2% Tween-20 and 5% skim milk and incubated overnight with antibody reported in Table 1. After several washes with PBS-Tween, the membranes were incubated for 45 min with the proper secondary antibody conjugated with horseradish peroxidase (1:2000, Dako, Italy). Immunoreactive bands were detected with enhanced chemiluminescence (ECL) reagents (Cyanagen, Bologna, Italy), according to the appropriate instructions, images were acquired by Azure 600 Imaging Systems. The density of the protein bands was normalized with the respective actin and subsequently with the loading control using ImageJ software.

### Statistical analyses

Analyses of mean value ± SEM were done using one-way ANOVA with Tukey test (GraphPad Prism 5.01). ρ values from ρ < 0.05 were considered statistically significant.

### Transmission electron microscopy (TEM) evaluations

Cells were processed for TEM analysis as previously described [12]. Briefly U251 cells were gathered by mild trypsinization (0.25% trypsin in PBS containing 0.05% EDTA) and collected by centrifugation at 800 rpm for 5 min in fresh tubes. The samples were fixed with 2.5% glutaraldehyde (Polysciences, Inc., Warrington, PA, United States) in culture medium for 2 h at room temperature, centrifuged at 1,000 rpm for 10 min, and washed in PBS. Samples were post-fixed in 1% OsO4 (Sigma Chemical Co., St. Louis, MO, United States) for 2 h at room temperature for lipid fixation and washed in water. The cell pellets were pre-embedded in 2% agar, and dehydrated with increasing concentrations of acetone (30, 50, 70, 90, and 100%, respectively). Finally, the pellets were embedded in Epon resin and polymerized at 60 °C for 48 h. Sections of 70–80 nm were cut on a Reichert OM-U3 ultramicrotome and collected on nickel grids (200 Mesh) and counterstained with uranyl acetate for 10 min and with lead citrate for 3 min. The specimens were observed with a JEM 1200 EX II (JEOL, Peabody, MA, USA) electron microscope operating at 100 kV and equipped with a MegaView G2 CCD camera (Olympus OSIS, Tokyo, Japan).

## Results

Based on the previous results, relating to the oxidative stress condition and the activation of the ferroptotic pathway, which emerged from the study of the effect of Micotherapy U-Care, administered in combination with Cisplatin and Pt(IV)Ac-POA, on the U251 cell line, in this work we paid attention to the different cell death mechanisms that can be triggered by the synergic action of those same actives.

### Long term efficacy of the treatments

**Fig. 1:**
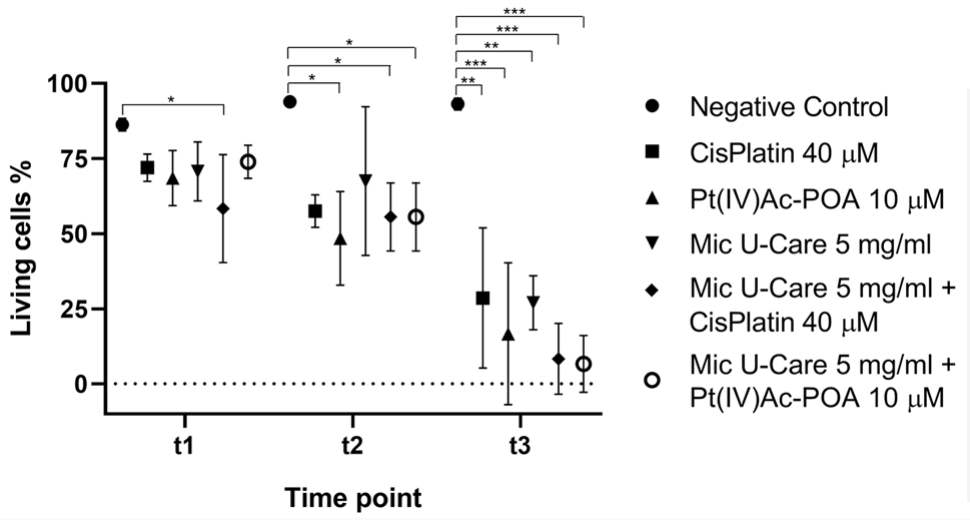
Graph representing the percentage, with respect to total cells, of viable U251 cells after the exposure to Cisplatin 40 μM, Pt(IV)Ac-POA 10 μM, Mic U-Care + Cisplatin and Mic U-Care + Pt(IV)Ac-POA after 2 h (t1) and 48 h (t2) of treatments and after the recovery period (t3). Statistical significance expressed as ****p < 0.0001; ***p < 0.001; **p < 0.01; *p < 0.1

The Trypan Blu-exclusion method reviled (Fig. 1) that among the different treatments taken alone, the mean percentage viability in the sample treated with Pt(IV)Ac-POA was found to be minimum at every time point with mean value respectively of 67.42% (± 1.10%, range 68.52–66.33%, ρ < 0.0001 vs. CTR, ρ = 0.0068 vs. Cisplatin, ρ = 0.0007 vs. Mic U-Care) at t1, 50.08% (± 1.67%, range 48.41–51.75%, ρ < 0.0001 vs. CTR, ρ = 0.9908 vs. Cisplatin, ρ < 0.0001 vs. Mic U-Care) at t2, and 28.33% (± 0.56%, range 27.78–28.89%, ρ < 0.0001 vs. CTR, ρ = 0.0001 vs. Cisplatin, ρ < 0.0001 vs. Mic U-Care) at t3. While among the combined treatments that of Mic U-Care + Pt(IV)Ac-POA was found to be minimum after 2 and 48 h of continuous exposure, i.e. 60.93% (± 4.91%, range 65.83–56.02%, ρ < 0.0001 vs. CTR, ρ = 0.0446 vs. Pt(IV)Ac-POA, ρ < 0.0001 vs. Mic U-Care, ρ = 0.077 vs. Cisplatin + Mic U-Care) at t1, 43.97% (± 2.86%, range 46.83–41.11%, ρ < 0.0001 vs. CTR, ρ = 0.0277 vs. Pt(IV)Ac-POA, ρ < 0.0001 vs. Mic U-Care, ρ = 0.0301 vs. Cisplatin + Mic U-Care) at t2. But the lowest mean percentage viability overall was given by Mic U-Care + Pt(IV)Ac-POA after the recovery period of 7 days, 25.65% (± 1.20%, range 24.44–26.85%, ρ < 0.0001 vs. CTR, ρ = 0.1813 vs. Pt(IV)Ac-POA, ρ < 0.0001 vs. Mic U-Care, ρ = 0.0054 vs. Cisplatin + Mic U-Care). Hight statistical validity of changes in cells’ viability over time (ρ < 0.0001) was revealed for each condition, in the case of Micotherapy U-Care + Pt(IV)AcPOA in t1 vs. t2 and t2 vs.t3 the respective ρ Value were 0.0004 and 0.0003.

### Impaired migration capability

**Fig. 2:**
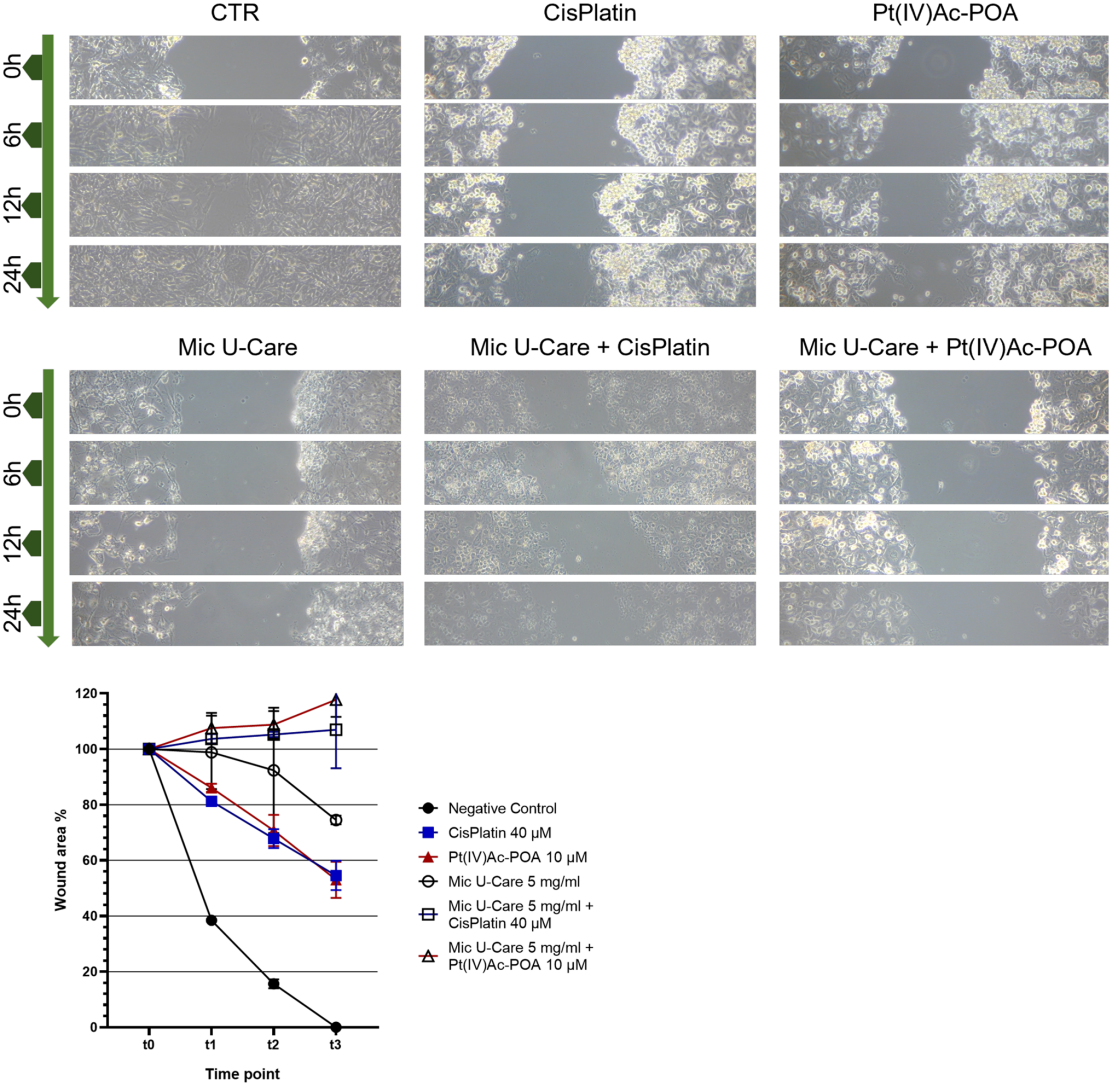
Wound healing scratch assay on non-treated and treated U251 cells. Graph showing the extent of scratch closure expressed as the percentage, with respect to the initial scratch area (t0), of open scratch are at 6 h (t1), 12 h (t2) and 24 h (t3)

Through the wound healing assay (Fig. 2), it emerged that if in 24 h the monolayer of untreated U251 cells completely closes (ρ < 0.0001 vs. CTR t0), the percentage of wounded area (W%) with respect to t0 of the platinum compounds used alone is slightly higher that 50%, respectively 53.01% ± 6.47% (ρ < 0.0001 vs. Cisplatin t0, ρ < 0.0001 vs. CTR t3 and ρ = 0.9998 vs Pt(IV)Ac-POA t3) for Cisplatin and 54.56% ± 5.26% (ρ < 0.0001 vs. Pt(IV)Ac-POA t0, ρ < 0.0001 vs. CTR t3 and ρ = 0.9998 vs Cisplatin t3) for Pt(IV)Ac-POA.

A non-statistically significant improvement emerged following the use of Micotherapy U-Care only with a W% equal to 74.50 ± 1.58 (ρ = 0.1565 vs. Mic U-Care t0, ρ < 0.0001 vs. CTR t3).The combined therapy has given promising results, the W% has not decreased with the passage of time, but has slightly increased, in particular the combined treatment of Cisplatin and the supplement reached in 24 h a W% of 117.75% ± 6.18% (ρ = 0,6332 vs. Cisplatin + Mic U-Care t0), while its equivalent with Pt(IV)Ac-POA of 106.96% ± 13.85% (ρ = 0,0083 vs. Pt(IV)Ac-POA + Mic U-Care t0). On one hand, these results seemed significant in comparison with the therapies taken individually at t3 (ρ ranging 0.0012–< 0.0001) but no significance was recorded when comparing the two combined treatments (ρ = 0.4661).

### Differentiated activation of apoptotic pathways

**Fig. 3:**
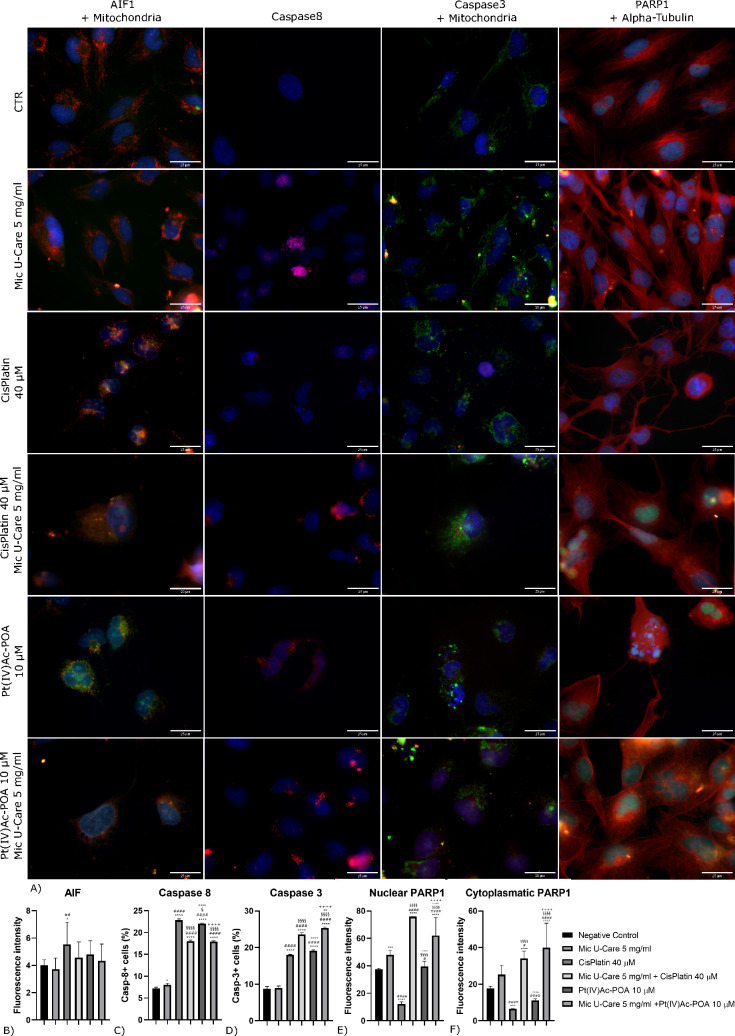
Immunolabeling for AIF1 (in green) and mitochondria (in red), Clieved Caspase 8 (in red), Clieved Caspase 3 (in green) and mitochondria (in red), PARP1 (in green) and Alpha Tubulin (in red): in the controls, differently treated U251 cells, i.e., after 48 h-CT with Micotherapy U-Care 5 mg/ml, with CDDP 40 μM, with Pt(IV)Ac-POA 10 μM, with Mic U-Care + CDDP, with Mic U-Care + Pt(IV)Ac-POA. DNA was stained with Hoechst 33,258 (blue fluorescence). Magnification 60X, bar of 25 µm. The histograms below show the fluorescence intensity value of the immunolabeling. Statistical significance calculated as follows: *control vs. each experimental condition; #Mic U-Care vs. other treatments; §Cisplatin vs. Pt(IV)Ac-POA and each combined treatment; °Cisplatin + Mic U-Care vs. Pt(IV)Ac-POA and Pt(IV)Ac-POA + Mic U-Care; + Pt(IV)Ac-POA vs. Pt(IV)Ac-POA + Mic U-Care. ****p < 0.0001. ***p < 0.001; **p < 0.01; *p < 0.1 (Color figure online)

As shown in Fig. 3A and in Supplementary material 1, in which a doble-immunolabeling of AIF, apoptotic inducing factor, and mitochondria is reported, mitochondria presented a homogenous distribution within the cytoplasm in the control, upon 48 h exposure to micotherapy 5 mg/ml, Cisplatin 40 µM and Pt(IV)Ac-POA 10 µM used alone their shape resulted more elongated. In combined therapy, with Cisplatin 40 µM + Mic U-Care 5 mg/ml and Pt(IV)Ac-POA 10 µM + Mic U-Care 5 mg/ml, looking mitochondria reduced in size, features of drug resistance weren’t detectable. Moreover, it was observable an accumulation of these organelles in the nuclear periphery in the samples exposed to chemotherapy alone, probably due to a more collapsed cell structure.

AIF labeling appeared colocalized with mitochondria in all the treatments, even if after combined therapy with the mushroom mixture and chemotherapy the staining appeared diffuser also involving the nuclear region. Quantifying the fluorescence intensity of AIF staining (Fig. 3B), a slight decrease was detectable in cells treated with Mic U-Care 5 mg/ml compared to control, similarly, the protein appeared less detectable, albeit in a non-statistically significative way, in samples treated with the combined therapy compared to the corresponding chemotherapy used alone. (Mic U-Care -7.08%, SEM 0.25, Cisplatin + 38.27%, SEM 0.49 (ρ = 0.0213), Cisplatin + Mic U-Care + 14.01%, SEM 0.35, Pt(IV)Ac-POA + 20.15%, SEM 0.30 and Pt(IV)Ac-POA + Mic U-Care + 8.37%, SEM 0.37 (all vs. CTR)).

The analysis of cleaved Caspase 8 (Fig. 3C, Supplementary material 2A) indicated that samples exposed to Mic U-Care 5 mg/ml showed a mild growing number of positive cells for Casp 8, those subjected to CDDP 40 μM or Pt(IV)Ac-POA 10 μM revealed an important increase of labelled cells suggesting the activation of the extrinsic apoptotic pathway. Contrary, the combined therapy resulted less efficient in the activation of this same cell death via, being a reduction of the cells positive to the staining compared to the corresponding chemotherapy used alone. (Mic U-Care + 0.8%, SEM 0.21 (ρ = 0.0295), Cisplatin + 15.64%, SEM 0.17 (ρ < 0.0001), Cisplatin + Mic U-Care + 10.82%, SEM 0.15 (ρ < 0.0001), Pt(IV)Ac-POA + 14.87%, SEM 0.07 (ρ < 0.0001) and Pt(IV)Ac-POA + Mic U-Care + 10.73%, SEM 0.10 (ρ < 0.0001) (all vs. CTR)) These data were confirmed by western blot analysis (Supplementary material 2B and 2C).

The analysis of cleaved Caspase 9 trough western blot (Supplementary material 2D and 2E) indicated a major increase in the cleaved form of 37 kDa and 17 kDa following chemotherapy combined with micotherapy compared to therapy with Cisplatin alone or fourth generation derivate.

To verify the activation status of the apoptotic pathway we also analyzed the expression of Caspase 3 (Fig. 3D, Supplementary material 3). The treatment with the only micotherapy induced a minor increase in apoptotic cells numbers, those with the chemotherapy alone and with the combined therapy pointed out an evident growing population of cell expressing Casp 3, in particular, a rise of Caspase 3-positive cells emerged in the samples exposed to the combined therapy in comparison to the those exposed to the matching chemotherapy alone. (Mic U-Care + 0.17%, SEM 0.32, Cisplatin + 9.30%, SEM 0.05 (ρ < 0.0001), Cisplatin + Mic U-Care + 14.87%, SEM 0.27 (ρ < 0.0001), Pt(IV)Ac-POA + 10.36%, SEM 0.13 (ρ < 0.0001) and Pt(IV)Ac-POA + Mic U-Care + 16.62%, SEM 0.16 (ρ < 0.0001) (all vs. CTR)).

Activated Caspase 3 acts by cleaving poly-(ADP-ribose)-polymerase 1 (PARP 1), involved in repair mechanisms upon DNA damage, in Fig. 3A and Supplementary material 4 is reported the immunolabeling of PARP 1 and alpha-tubulin. The tubulin cytoskeleton showed the typical astrocytic-like structure in the untreated cells, this feature was loosed in the sample treated with Mic U-Care 5 mg/ml, in which cells looked more streamlined; the cells exposed to chemotherapy took a collapsed tubular morphology, that was anormal in the samples subjected to combined therapy too.

Our founding suggested that PARP 1 localized both in the nucleus and in the cytoplasm in control cells and in cells exposed to the micotherapy alone, after exposure to CDDP 40 μM or Pt(IV)Ac-POA 10 μM PARP1 levels decreased in all the cellular compartment, but in particular in the cytoplasm compared to the nucleus. Conversely, PARP1 was more abundant in the nucleus and cytoplasm of the cells exposed to Micotherapy U-Care 5 mg/ml in combination whit the chemotherapy (Fig. 3E, F). Particularly, in our samples treated with Cisplatin 40 μM + Mic U-Care 5 mg/ml or Pt(IV)Ac-POA 10 μM + Mic U-Care 5 mg/ml, PARP 1 stood in perinuclear spots, probably the mitochondria, where it interacts with mitochondrial BER enzymes, impairing the repair of the oxidative-induced damage to the mitochondrial DNA. (In the nuclear region: Mic U-Care + 27.98%, SEM 0.92 (ρ = 0.008), Cisplatin -67.52%, SEM 0.48 (ρ < 0.0001), Cisplatin + Mic U-Care + 102.27%, SEM 0.04 (ρ < 0.0001), Pt(IV)Ac-POA + 5.83%, SEM 1.10 (ρ = 0.9449) and Pt(IV)Ac-POA + Mic U-Care + 65.84%, SEM 3.91 (ρ < 0.0001).

In the cytoplasm: Mic U-Care + 41.62%, SEM 1.52 (ρ = 0.0610), Cisplatin -62.82%, SEM 0.04 (ρ = 0.0008), Cisplatin + Mic U-Care + 91.69%, SEM 1.21 (ρ < 0.0001), Pt(IV)Ac-POA -37.73%, SEM 0.29 (ρ = 1140) and Pt(IV)Ac-POA + Mic U-Care + 125.05%, SEM 4.01 (ρ < 0.0001) (all vs. CTR)).

### Autophagic pathway and mitophagy are not strictly mutual

**Fig. 4:**
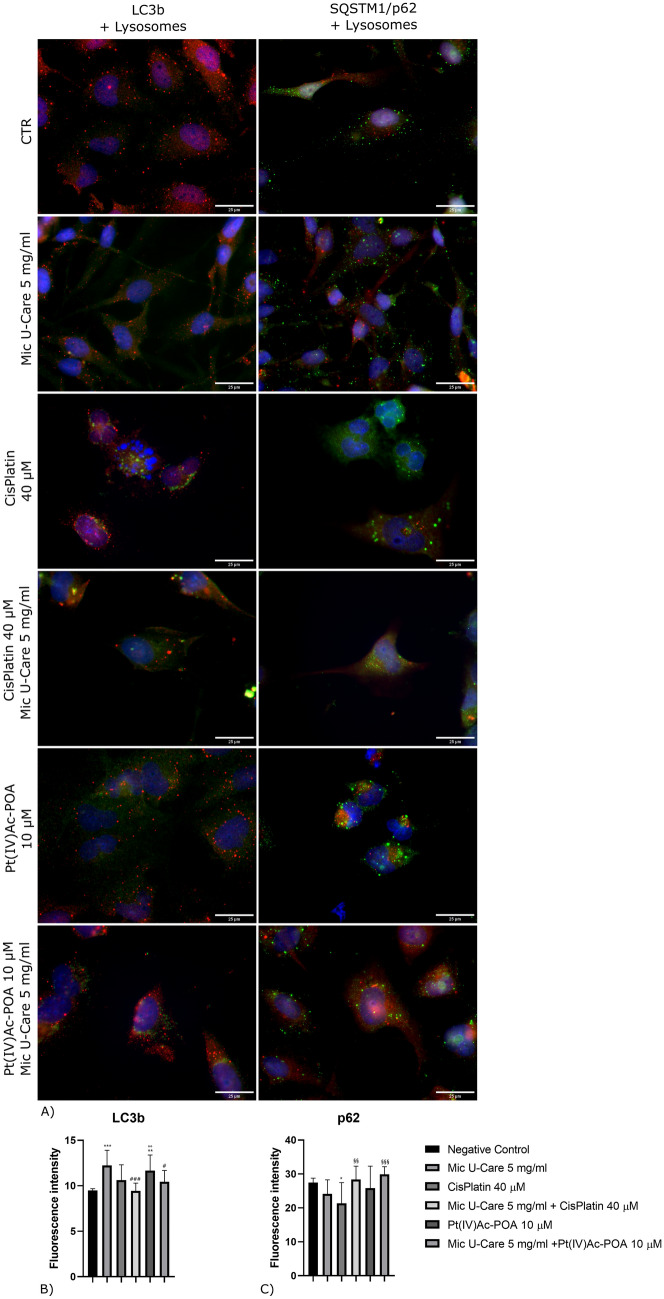
Immunolabeling for LC3b (in green) and lysosomes (in red), SQSTM/p62 (in green) and lysosomes (in red): in the controls, differently treated U251 cells, i.e., after 48 h-CT with Micotherapy U-Care 5 mg/ml, with CDDP 40 μM, with Pt(IV)Ac-POA 10 μM, with Mic U-Care + CDDP, with Mic U-Care + Pt(IV)Ac-POA. DNA was stained with Hoechst 33,258 (blue fluorescence). Magnification 60X, bar of 25 µm. The histograms below show the fluorescence intensity value of the immunolabeling. Statistical significance calculated as follows: *control vs. each experimental condition; #Mic U-Care vs. other treatments; §Cisplatin vs. Pt(IV)Ac-POA and each combined treatment; °Cisplatin + Mic U-Care vs. Pt(IV)Ac-POA and Pt(IV)Ac-POA + Mic U-Care; + Pt(IV)Ac-POA vs. Pt(IV)Ac-POA + Mic U-Care. ****p < 0.0001. ***p < 0.001; **p < 0.01; *p < 0.1 (Color figure online)

To deepen the investigation inherent the different cell death pathways we studied the activation of autophagy evaluating LC3B and SQSTM 1/p62, reported in a doble-immunolabeling with lysosomes in Fig. 4A, Supplementary material 5 and 6.

In control samples lysosomes were homogeneously distributed throughout the cytoplasm, although after the treatment with the different compounds they seemed to increase in number; moreover, after the therapy with Cisplatin 40 μM or Pt(IV)Ac-POA 10 μM alone and in combination with Mic U-Care 5 mg/ml they looked bigger.

LC3B, involved in autophagosome formation, appeared equally distributed in the cytoplasm of the control sample, similarly in the ones treated with Mic U-Care, Cisplatin + Mic U-Care and Pt(IV)Ac-POA. Its staining assumed a dotted shape neatly colocalizing with lysosomes in the cells that had been given CDDP 40 µM, while, although there were marking spots in the Pt(IV)Ac-POA 10 μM + Mic U-Care 5 mg/ml treated, the colocalization with lysosomes didn’t occur. We detected an increasing of LC3B expression in the cells exposed to the single compound, (Fig. 4B) while the level of the protein decreased after the treatment with the combined therapies compared to the respective chemotherapy used alone, trend confirmed in western blot analysis (Supplementary material 5B and 5C). (Mic U-Care + 28.92%, SEM 0.53 (ρ = 0.0003), Cisplatin + 11.81%, SEM 0.51, Cisplatin + Mic U-Care -0.573%, SEM 0.27, Pt(IV)Ac-POA + 22.91%, SEM 0.52 (ρ = 0.0047) and Pt(IV)Ac-POA + Mic U-Care + 9.95%, SEM 0.39 (all vs. CTR)).

As for LC3B, SQSTM1/p62 scaffold protein is homogeneously distributed in the cytoplasm in all the samples, except for those subjected to the only chemotherapy, in which it was spotted-like in the perinuclear area. Its level of expression (Fig. 4C, Supplementary material 6B and 6C) was lower in those cells treated with Mic U-Care 5 mg/ml, Cisplatin 40 μM or Pt(IV)Ac-POA 10 μM administered alone, while an increasing was detectable in the samples treated with combined therapy of CDDP + Mic U-Care or Pt(IV)Ac-POA + Mic U-Care in comparison to the corresponding chemotherapy alone. (Mic U-Care -12.06%, SEM 1.32, Cisplatin -22.248%, SEM 1.85 (ρ = 0.0277), Cisplatin + Mic U-Care + 3.44%, SEM 1.25, Pt(IV)Ac-POA -5.97%, SEM 1.60 and Pt(IV)Ac-POA + Mic U-Care + 8.855%, SEM 0.73 (all vs. CTR)).

**Fig. 5:**
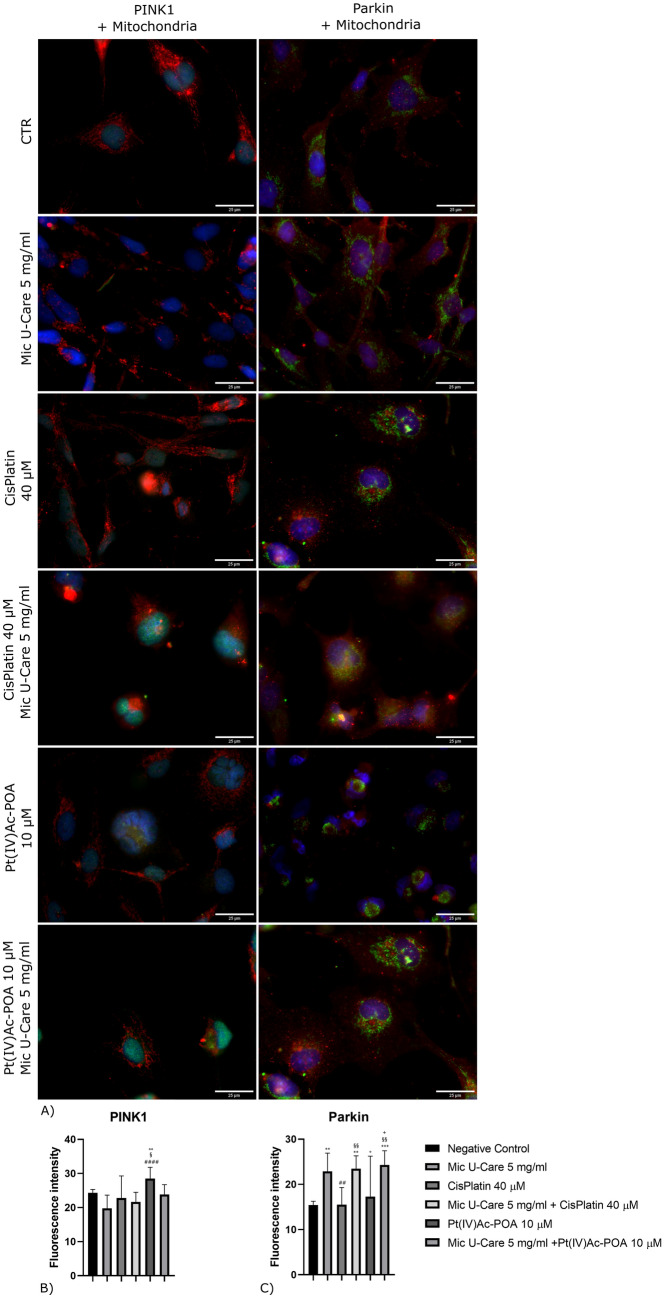
Immunolabeling for PINK1 (in green) and mitochondria (in red), Parkin (in red) and mitochondria (in green): in the controls, differently treated U251 cells, i.e., after 48 h-CT with Micotherapy U-Care 5 mg/ml, with CDDP 40 μM, with Pt(IV)Ac-POA 10 μM, with Mic U-Care + CDDP, with Mic U-Care + Pt(IV)Ac-POA. DNA was stained with Hoechst 33,258 (blue fluorescence). Magnification 60X, bar of 25 µm. The histograms below show the fluorescence intensity value of the immunolabeling. Statistical significance calculated as follows: *control vs. each experimental condition; #Mic U-Care vs. other treatments; §Cisplatin vs. Pt(IV)Ac-POA and each combined treatment; °Cisplatin + Mic U-Care vs. Pt(IV)Ac-POA and Pt(IV)Ac-POA + Mic U-Care; + Pt(IV)Ac-POA vs. Pt(IV)Ac-POA + Mic U-Care. ****p < 0.0001. ***p < 0.001; **p < 0.01; *p < 0.1 (Color figure online)

In order to investigate the autophagic pathway, the activation of mitophagy was also evaluated using PINK 1, a mitochondrial damage signal, and Parkin as markers, the results are reported in Fig. 5, in which the doble-immunolabeling with mitochondria are showed.

PINK1 labeling (Supplementary material 7) is diffuse in all the sample, although after treatments with Cisplatin 40 μM or Pt(IV)Ac-POA 10 μM and their combination with Mic U-Care 5 mg/ml, a stronger localization of the fluorescence at the level of mitochondria was observed. In our samples, PINK 1 was overexpressed in particular after treatment with Pt(IV)Ac-POA 10 μM, (Fig. 5B) to note that just as after the administration of the micotherapy alone the cells showed a lower PINK 1 marking, similarly, after the treatment with the combined therapy there was a decrease in fluorescence compared to the respective chemotherapy used alone. (Mic U-Care -18.66%, SEM 1.22, Cisplatin -6.20%, SEM 1.95, Cisplatin + Mic U-Care -10.98%, SEM 0.90, Pt(IV)Ac-POA + 17.22%, SEM 1.00 and Pt(IV)Ac-POA + Mic U-Care -2.07%, SEM 0.92 (all vs. CTR)).

As seen previously, Parkin immunolabeling (Supplementary material 8) and its modification after the different treatments were coherent with what observed for PINK 1, indeed, Parkin localization appeared strongly associated with mitochondria after the treatment with the chemotherapy and the combined therapy, while widespread in the control sample and in the treated with Mic U-Care only. Parkin levels (Fig. 5C) increased in cells after every treatment, particularly after the exposition to Micotherapy U-Care alone and as an adjuvant in combination with CDDP or Pt(IV)Ac-POA. (Mic U-Care + 48.37%, SEM 1.28 (ρ = 0.0077), Cisplatin + 0.60%, SEM 1.15, Cisplatin + Mic U-Care + 52.49%, SEM 0.89 (ρ = 0.0030),

Pt(IV)Ac-POA + 12.11%, SEM 2.70 and Pt(IV)Ac-POA + Mic U-Care + 57.59%, SEM 0.99 (ρ = 0.0009) (all vs. CTR)).

### RIPK1 independent activation of programmed necrosis

**Fig. 6:**
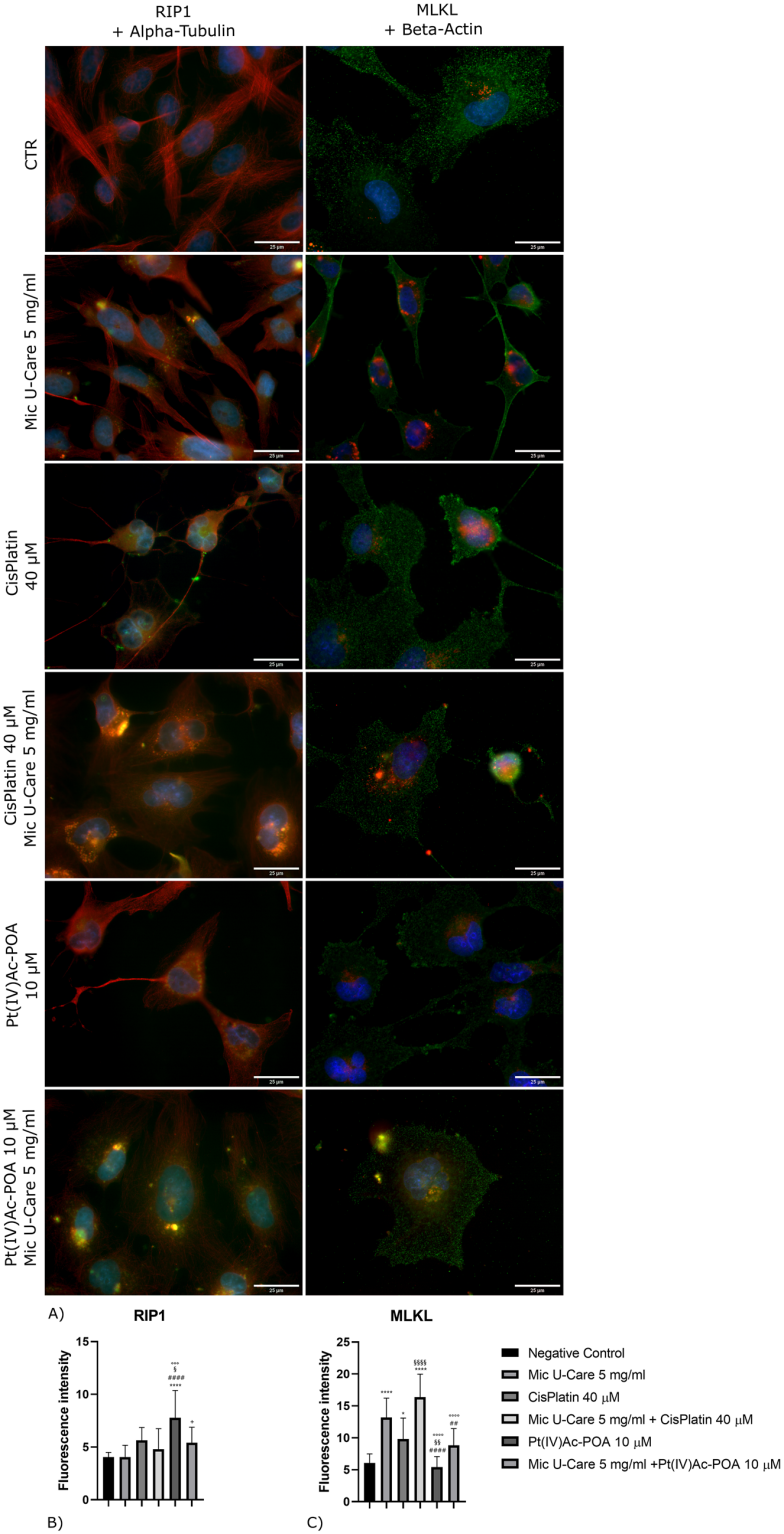
Immunolabeling for RIP1 (in green) and Alpha tubulin (in red), MLKL (in red) and Beta Actin (in green): in the controls, differently treated U251 cells, i.e., after 48 h-CT with Micotherapy U-Care 5 mg/ml, with CDDP 40 μM, with Pt(IV)Ac-POA 10 μM, with Mic U-Care + CDDP, with Mic U-Care + Pt(IV)Ac-POA. DNA was stained with Hoechst 33,258 (blue fluorescence). Magnification 60X, bar of 25 µm. The histograms below show the fluorescence intensity value of the immunolabeling. Statistical significance calculated as follows: *control vs. each experimental condition; #Mic U-Care vs. other treatments; §Cisplatin vs. Pt(IV)Ac-POA and each combined treatment; °Cisplatin + Mic U-Care vs. Pt(IV)Ac-POA and Pt(IV)Ac-POA + Mic U-Care; + Pt(IV)Ac-POA vs. Pt(IV)Ac-POA + Mic U-Care. ****p < 0.0001. ***p < 0.001; **p < 0.01; *p < 0.1 (Color figure online)

To identify a possible involvement of the necroptotic pathway in the decreasing of cellular density observed after the treatment with the two chemotherapies in study and their combination with Micotherapy U-Care, the localization and the level of expression of RIP 1 and MLKL, involved in the formation of the necrosome, were evaluated (Fig. 6).

Microscopy images indicated that RIP 1 labeling (Supplementary material 9) was weak and homogeneous in the cytosol in the control condition, while after the different treatments it appeared more spot-like. In particular, bigger dots appeared in the perinuclear area in the cells exposed to Pt(IV)Ac-POA 10 μM and Cisplatin 40 μM or Pt(IV)Ac-POA 10 μM in combination with Mic U-Care 5 mg/ml, moreover, a major nuclear labelling of this protein was observable after the treatments with micotherapy. The expression of RIP 1 (Fig. 6B) increased in samples treated with chemotherapy alone and in combination with micotherapy, but mostly in those exposed to the only chemotherapy, most of all after Pt(IV)Ac-POA 10 μM. (Mic U-Care + 0.16%, SEM 0.34, Cisplatin + 39.36%, SEM 0.36 (ρ = 0.1736), Cisplatin + Mic U-Care + 18.75%, SEM 0.59, Pt(IV)Ac-POA + 92.28%, SEM 0.78 (ρ < 0.0001) and Pt(IV)Ac-POA + Mic U-Care + 33.87%, SEM 0.44 (all vs. CTR)).

MLKL is an effector protein that acts downstream of RIPK 1, in our untreated samples its staining appeared spread in the cytosolic compartment, indeed, only few cells showed a dotted coloring. After the treatments it was noticeable a more intense labeling assuming spot-like shape in the perinuclear area, probably in correspondence whit vesicles related to the Golgi apparatus. Having performed a double immunolabeling (Supplementary material 10) with Beta-actin too, in our samples, it was evident that actin cytoskeleton was well organized in control, although, it undergone to structural changes in the cells exposed to the micotherapy, cells in this sample appeared more elongated with compressed nuclei. After the exposure to CDDP 40 µM or Pt(IV)Ac-POA 10 µM was noticeable a collapsed cytoskeleton morphology but filipodia were still visible, while after the combined therapies actin distribution appeared looser. MLKL fluorescence intensity (Fig. 6C) increased more significantly in the samples subjected to therapy with micotherapy and micotherapy in combination with the chemotherapy drugs. (Mic U-Care + 118.05%, SEM 0.92 (ρ < 0.0001), Cisplatin + 62.51%, SEM 0.99 (ρ = 0.0222), Cisplatin + Mic U-Care + 170.97%, SEM 1.09 (ρ < 0.0001), Pt(IV)Ac-POA -10.44%, SEM 0.50 and Pt(IV)Ac-POA + Mic U-Care + 45.94%, SEM 0.79 (ρ = 0.1766) (all vs. CTR)).

### Transmission electron microscopy confirmation

**Fig. 7:**
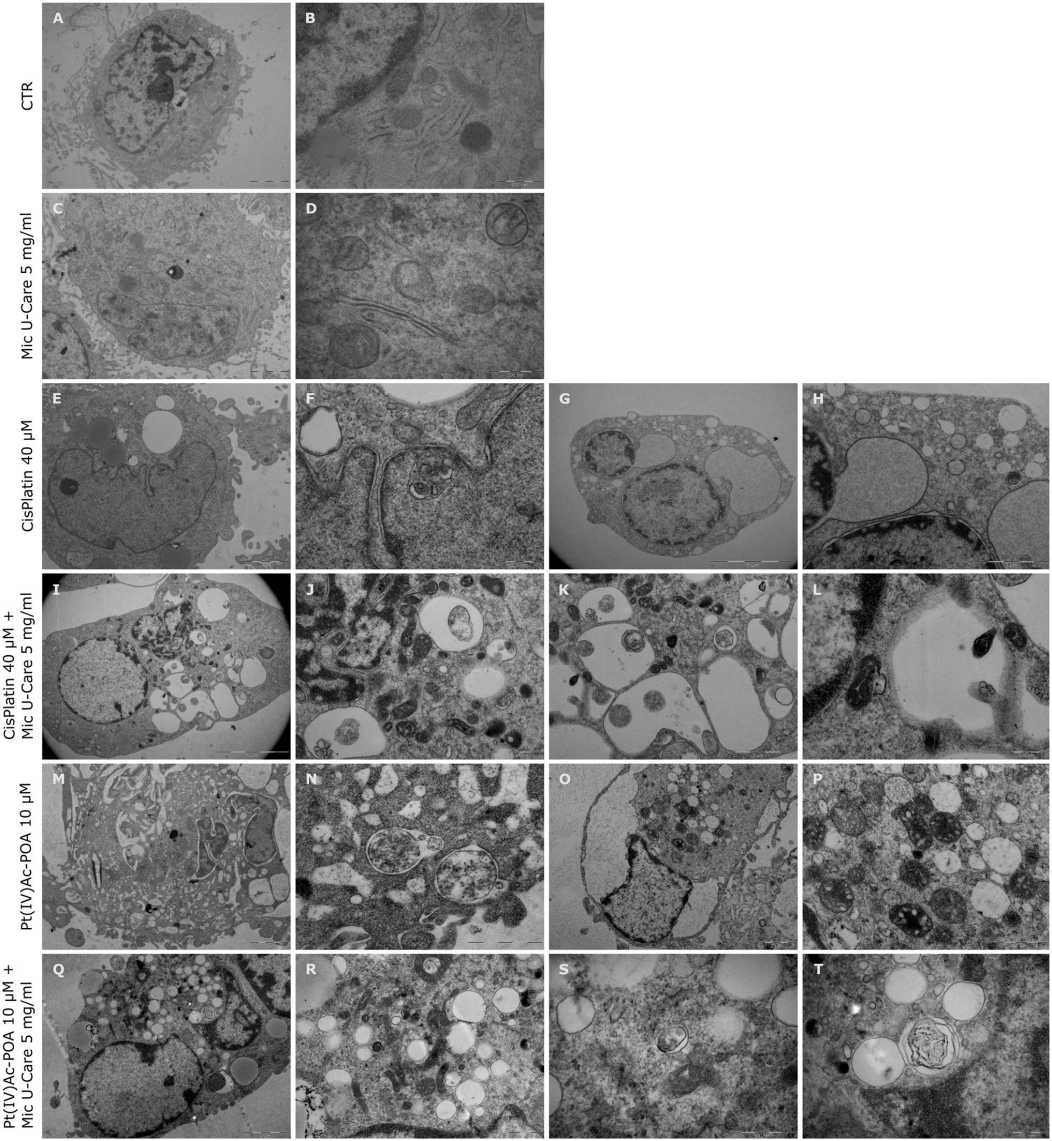
Ultrastructural analysis by transmission electron microscopy (TEM). Magnifications: X10k (**A**, **C**, **M**, **O**), X50k (**B**, **F**, **L**, **T**), X60k (**D**), X12k (**E**, **Q**), X7.5 k (**G**, **I**), X20k (**H**, **J**), X30k (**K**, **P**, **R**), X40k (**N**), X75k (**S**) Bars: 2 μm (**A**, **C**, **E**, **H**, **K**, **M**, **O**, **Q**), 500 nm (**B**, **D**, **F**, **L**, **S**, **T**), 5 μm (**G**, **I**), 1 μm (**J**, **N**, **P**, **R**)

Transmission electron microscopy micrographs were reported in Fig. 7. In the control sample nucleus was well organized: the intact nuclear envelope, the state of chromatin condensation and the nucleolus along with nuclear bodies, were distinguishable (Fig. 7A). In the cytoplasm of untreated in cells, a well-defined rough endoplasmic reticulum (RER) was present, as the oval profiles of healthy mitochondria were observable (Fig. 7B). The photographs of the cells treated with Mic U-Care 5 mg/ml showed a less defined nuclear lamina in some portions, similarly the electron dense structures corresponding to nuclear bodies were no longer appreciable (Fig. 7C). Changes were also noticeable in the cytoplasm, such as loss of RER and the presence of more translucent mitochondria with deformed inner-membrane cristae (Fig. 7D); those are signs of damage induced by oxidative stress and reduced synthetic activity. Ultrastructural analysis revealed the coexistence of cells bearing the characteristics of autophagic and necroptotic processes in the samples treated with chemotherapy alone, Cisplatin 40 µM or Pt(IV)Ac-POA 10 µM.

Some cells, such as those shown in Fig. 7E–F and M–N, showed, indeed, normal nucleus with, at most, partially decondensed chromatin, swollen mitochondria and double membranes vesicles, probably autophagosomes. On the other hand, there were, as in Fig. 7G–H and O–P, cells showing a morphology typical of cells in necroptosis: disappearance of the nucleosome, condensed chromatin relegated to the nuclear membrane, dilated perinuclear space from the detachment of the nuclear lamina, decreasing of cytoplasmatic electron-density, swollen organuli and increasing of vacuolization. The images relating to the samples treated in combined therapy (Fig. 7I–L and Q–T, instead, show typical signs of mitophagy and ferroptosis. In the same cell, there were observable isolation membranes with mitochondria enclosed in them and small abnormal mitochondria, which define the process of mitophagy, it could also be noticed the presence of swollen mitochondria more electron-dense and with destroyed cristae, translucid nucleus with lateral condensed chromatin and electron lucent cytoplasm, feature of ferroptotic death mechanism. Moreover, in Fig. 7T a multilamellar body, lysosomal organelle, typical of suffering cell, is reported.

## Discussion

In this work we wanted to verify if the combined treatment of fungal-based supplements, as Micotherapy U-Care endowed of antioxidant and antitumoral properties, and platinum-based chemotherapy could represent a valid therapy against Glioblastoma Multiforme. Having obtained promising results about a more accurate control of the cellular redox state that did not impair on the initialization of the ferroptotic pathway, here we evaluated the different cell death mechanisms that can be triggered by the synergic action of micotherapy and chemotherapy, with the hope of developing a new combination therapy to improve the quality of life and the patient survival expectations. Our results have highlighted how the new fourth generation molecule, Pt(IV)Ac-POA, is more efficient in the long term than the already introduced in clinic Cisplatin. Moreover, the combined therapy of both chemotherapy with the fungal supplement was particularly promising following the recovery period, which could simulate the interval between one chemotherapy cycle and the next. The ability of tumor cells to migrate, a point that raises great difficulties in the effectiveness of currently used treatments, also appears to be reduced following the administration of the combination therapy.

The conducted experiments revealed the induction in the U251 cells of morphological changes by the different treatments. Mitochondria assumed an elongated shape after the exposure to the micotherapy or the chemotherapy alone, typical feature of drug resistance [13], this same characteristic wasn’t detectable after the administration of the combined therapies of Cisplatin 40 µM + Mic U-Care 5 mg/ml or Pt(IV)Ac-POA 10 µM + Mic U-Care 5 mg/ml, suggesting an implication of the synergic effects of the two classes of drug in the curbing of the resistant mechanism onset in the cell line, naturally resistant to chemotherapy, here considered. Moreover, after the treatment with micotherapy used together chemotherapy mitochondria seemed reduced in size, possible sign of cellular distress. Observing the lysosomes condition, it could be possible to verify a change in their morphology and numerosity, the former saw an increase in the size of these organelles after the treatment with Cisplatin 40 μM or Pt(IV)Ac-POA 10 μM alone and in combination with Mic U-Care 5 mg/ml, the letter because after the treatment with the different compounds they seemed to increase in number. Furthermore, if untreated cells showed an astrocytic structure, those subjected to the micotherapy appeared more elongated with flattened nuclei, while those exposed to the chemotherapy alone and in combination with the micotherapy seemed with a collapsed cytoskeleton morphology.

From the studies carried out it emerged that the apoptotic pathway was successfully activated by the use of chemotherapy alone or with the combined therapy. The apoptotic pathway can be mediated by AIF (Apoptosis-Inducing Factor), a flavoprotein with NADH-oxidase activity with redox function within the mitochondria in which it is normally located. The increase in mitochondrial permeability following apoptotic signals results in the release of apoptogenic effectors, such as AIF, which is released into the cytosol and subsequently transported to the nucleus. AIF interacts directly with nuclear DNA and leads to chromatin condensation, large-scale DNA fragmentation and cell death [14]. Even if in our samples, the expression of AIF was significantly increased only in cells treated only with Cisplatin, the nuclear localization of the protein was more evident in the samples subjected to combined therapy, suggesting a major mitochondrial disfunction induced by the synergic activity of chemotherapy and micotherapy that guarantee an increasing quantity of free AIF able to enter through the nuclear lamina. Indeed, in the treated samples a growing population of cells expressing the cleaved caspase 3 appeared, in particular, a rise of caspase 3-positive cells occurred in the samples exposed Cisplatin 40 μM or Pt(IV)Ac-POA 10 μM plus Micotherapy U-Care 5 mg/ml. Caspases are a class of cysteine-aspartic proteases that recognize aspartate residues on intracellular proteins, like other caspases. Caspase 3 is downstream of the apoptotic activation pathways, while caspase 8 is the initiator of the extracellular apoptotic pathway that is activated by death receptors on the plasma membrane [15]. According to the data obtained, the samples treated with chemotherapy alone showed a higher positivity to cleaved caspase 8, this founding and those previously discussed suggested that the apoptotic paths activated by the treatments here analyzed are different: after the chemotherapy alone, it appeared favorited the extrinsic one, while after the combined therapies of Mic U-Care and chemotherapy the mitochondrial one resulted more activated.

Among the substrates of caspase-3 there is PARP-1 (Poly-ADP-Ribose Polymerase-1), this protein has an N-terminal domain capable of binding DNA thanks to the presence of a double zinc structure, called DBD (DNA Binding Domain). DBD binds with high affinity to single- or double-stranded rupture sites, thus, in the presence of low levels of DNA damage, PARP-1 acts as a survival factor, while in presence of extensive damage acts to promote cell death. Usually, PARP 1 is sited in the nuclei in cells that showed a lightly damaged DNA to try and resolve the aberration, while it repositions in the cytoplasm in late apoptosis [16]. This protein according our studies appeared to be localized both at the nuclear and cytoplasmatic level in the samples; but an important decrease of PARP1 expression in the nucleus was preferentially recorded in the cells subjected to treatment with Cisplatin indicating that probably the DNA damage inflicted by this compound is lower than that caused by Pt(IV)Ac-POA alone and by combined chemotherapy to myotherapy. Moreover, an increment of PARP1 in the cytoplasmatic compartment was detected after the use of the combined therapies, indicating a probable more advanced apoptotic state. Particularly, in our samples treated with Cisplatin 40 μM + Mic U-Care 5 mg/ml or Pt(IV)Ac-POA 10 μM + Mic U-Care 5 mg/ml, PARP 1 stood in perinuclear spots, probably the mitochondria, where it interacts with mitochondrial BER enzymes, impairing the repair of the oxidative-induced damage to the mitochondrial DNA.

Using some marker of autophagy, such as LC3B and SQSTM 1/p62, it was observed that the combined therapy seems less effective in the activation of this pathway, which is a form of cell death, but can also be a survival strategy. The LC3 ubiquitin-like protein is cleaved at its C-terminus to form LC3B-I, which is then conjugated with phosphatidylethanolamine in the autophagosome membrane, promoting autophagy. LC3B-I is involved in autophagosome formation and interacts with another essential protein, SQSTM 1/p62, an ubiquitin-binding scaffold protein [17]. Both markers remained at baseline levels after treatment with combined therapy, while after the use of chemotherapy alone the inversely proportional trend, for which an increase in LC3b corresponds to a decrease in p62, typical of activation and completion of the autophagic flux, emerged. Thanks to these results we can hope that the use of micotherapy benefits a reduction of the adaptive response of tumor cells to the antitumor activity of the chemotherapies available.

While not having seen an activation of the autophagic pathway after the subjecting of Cisplatin 40 μM or Pt(IV)Ac-POA 10 μM in combination with Micotherapy U-Care 5 mg/ml, combined therapy, in particular with the fourth generation platinum compound, appeared to activate the mithophagic path successfully. This pathway was evaluated analyzing PINK 1 and Parkin expression, the PINK 1 protein (PTEN-induced kinase 1) has the physiological role of protecting cells from mitochondrial dysfunctions during stress conditions. In persisting condition of damaged mitochondria, changes of transmembrane potential cause the accumulation of PINK1 on the OMM (outer mitochondrial membrane), where it is activated by phosphorylation. Once activated, PINK1 is able to phosphorylate OMM proteins that will act as receptors for the binding of Parkin to the membrane, favoring the ubiquitination of mitochondrial proteins that will subsequently be degraded with mitophagy [18].In our samples, the Parkin levels increased in the cells treated with micotherapy alone and as an adjuvant localizing neatly with mitochondria, PINK 1 was overexpressed in particular after Pt(IV)Ac-POA but strongly associated with mitochondria in cells exposed the combined therapy with Mic U-Care.

Moreover, RIP 1 and MLKL proteins, both involved in the formation of the necrosome, essential for inducing necroptosis, were evaluated. The deubiquitinated form of the protein kinase RIP 1 (Receptor-Interacting Protein 1) into the cytoplasm constitutes a complex with also caspase 8. Caspase 8, in its active form, cleaves RIPK 1 inducing apoptosis; on the contrary, the inhibition of caspase 8 activity by FLIP, a catalytically inactive homolog of caspase 8 that can combine in complex II, can block the cleavage of RIPK 1 determining necroptosis [19]. In our samples, a significant increase in the level of RIP 1 was appraised only after treatment with Pt(IV)Ac-POA; this result could be consistent with the increase in caspase 8 found in all the samples subjected to each of the treatments under examination. Furthermore, fluorescence microscopy revealed a greater nuclear localization of RIP 1 in the cells undergoing combined therapies. Some studies report the fundamental role of RIP 1 at the nuclear level in the activation of PARs polymerases following excessive oxidative stress [20]. In support of this, as already said, an increase in total PARP levels was found following the combined administration of chemotherapy and micotherapy. An overactivation of PARs polymerases seems to be responsible for the mitochondrial depolarization and the consequent releasing of AIF, which can thus translocate into the nucleus and favor DNA fragmentation, as observed in our samples treated with combined therapy. These finds supported the activation of a mitochondrial dysfunction-PARP mediated form of cell death, the parthanatos, a regulated form of necrosis typical of an oxidative imbalance.

On the other hand, regarding the expression of MLKL, in our studies an increase of this protein was found in samples subjected to treatment with Mic U-Care 5 mg/ml alone or in combination with Cisplatin 40 μM or Pt(IV)Ac-POA 10 μM. MLKL, usually located in the cytosol, after being activated from RIPK 3 onto the necrosome, moves into vesicles of the Golgi apparatus and from there to the plasma membrane where it interacts with the phosphatidylinositol phosphate (PIP) triggering the final stage of necroptosis, which leads to the destruction of the membrane. RIPK 3 has been reported to mediate RIPK 1-independent necroptosis, but it remains unclear how to transduce necroptotic signals from TNFR 1 to RIPK 3 in the absence of RIPK 1 [21]. According to the data just discussed it was supposable that our combined treatments determined the activation of necroptosis, independently from RIP 1, but only an early necroptotic stage was findable. This indirect activation of the necroptotic pathway could be due to excessive mitochondrial stress [11] or to impaired capability of the treated cells to repair themselves. Moreover, the activation of this type of cell death could lead us to think that a specific immune response against cancer cells can be activated in a more complex living system.

The results presented show that both Cisplatin and Pt(IV)Ac-POA in combination with michoterapy are able to induce different patterns of cell death, such as apoptosis, mitophagy and regulated necrosis. To deepen the understanding of the mechanisms of adaptations that cancer cells put in place to preserve their survival, recovery conditions after continuous exposure to drugs will be investigated, hoping to identify specific inhibitors involved in these processes and new targets for the treatment of glioblastoma.

## Supplementary Information

Below is the link to the electronic supplementary material.

Supplementary file1 (DOCX 85042 KB)

## Data Availability

Data sharing not applicable to this article as no datasets were generated or analyzed during the current study.
