# Supplementary material for: Study on the activation of cell death mechanisms: in search of new therapeutic targets in glioblastoma multiforme
Source: Apoptosis. 2023 May 27;28(7-8):1241–57. doi: 10.1007/s10495-023-01857-x (PMC10333377; doi:10.1007/s10495-023-01857-x)
Supplement: Supplementary file 1 — Supplementary file1 (DOCX 85042 KB) [file 10495_2023_1857_MOESM1_ESM.docx]

**Study on the activation of cell death mechanisms: in search of new therapeutic targets in glioblastoma multiforme**

**Ludovica Gaiaschi^1^**
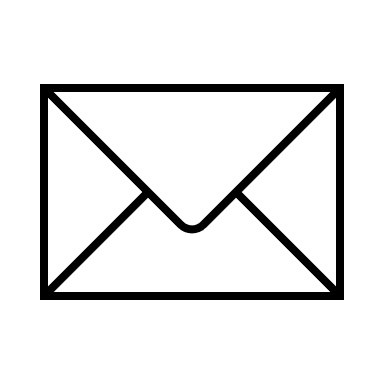
 **· Cristina Favaron^1^ · Claudio Casali^1^ · Federica Gola^1^ · Fabrizio De Luca^1^ · Mauro Ravera^2^ · Elisa Roda^3^ · Paola Rossi^4^ · Maria Grazia Bottone^1^**

^1^ Laboratory of Cell Biology and Neurobiology, Department of Biology and Biotechnology “L. Spallanzani”, University of Pavia, Via Ferrata 9, 27100 Pavia, Italy

^2^ Department of Sciences and Technological Innovation (DiSIT), University of Piemonte Orientale “A. Avogadro”, Viale Teresa Michel 11, 15121 Alessandria, Italy

^3^ Laboratory of Clinical and Experimental Toxicology, Pavia Poison Centre, National Toxicology Information Centre, Toxicology Unit, ICS Maugeri Spa, IRCCS Pavia, Via Maugeri 10, Pavia, Italy

^4^ Laboratory of Neurophysiology and Integrated Physiology, Department of Biology and Biotechnology “L. Spallanzani”, University of Pavia, Via Ferrata 9, 27100 Pavia, Italy

Ludovica Gaiaschi
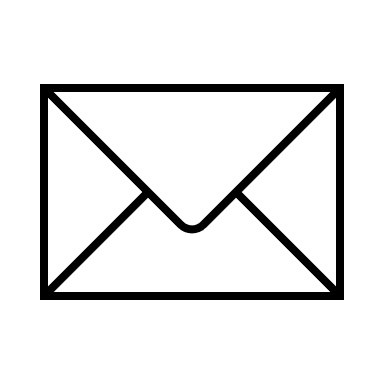

e-mail: ludovica.gaiaschi@unipv.it
ORCID: https://orcid.org/0000-0002-1880-2277

Cristina Favaron
e-mail: cristina.favaron01@universitadipavia.it

Claudio Casali
e-mail: claudio.casali@unipv.it

Federica Gola
e-mail: federica.gola01@universitadipavia.it

Fabrizio De Luca
e-mail: fabrizio.deluca@unipv.it

Mauro Ravera
e-mail: mauro.ravera@uniupo.it

Elisa Roda
e-mail: elisa.roda@unipv.it

Paola Rossi
e-mail: paola.rossi@unipv.it

Maria Grazia Bottone
e-mail: mariagrazia.bottone@unipv.it


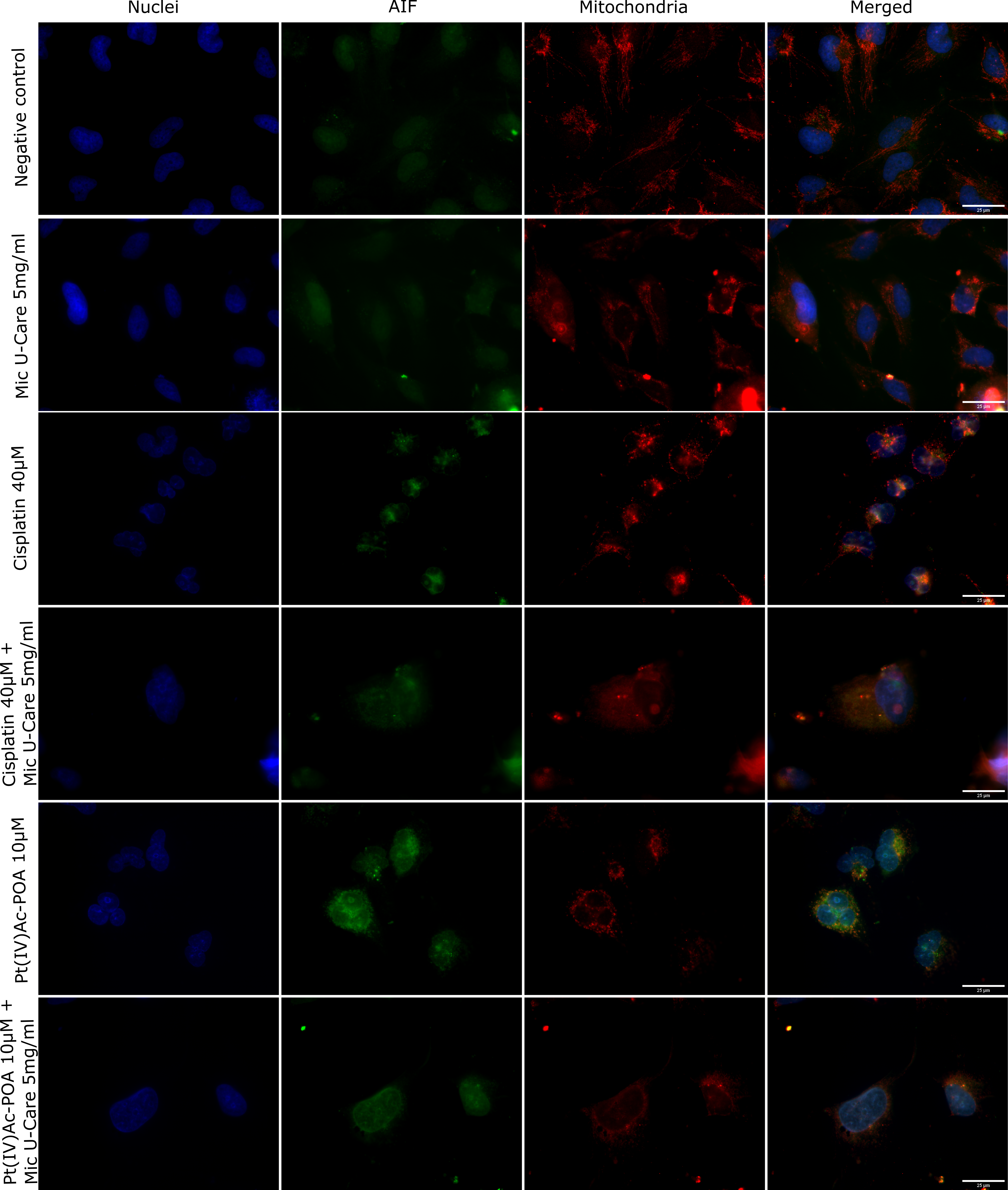


**Supplementary material 1** Immunolabeling for AIF1 (in green) and mitochondria (in red), DNA was stained with Hoechst 33258 (blue fluorescence), in U251 in control condition, and after 48 h-CT with Micotherapy U-Care 5 mg/ml, with CDDP 40 μM, with Pt(IV)Ac-POA 10 μM, with Mic U-Care + CDDP, with Mic U-Care + Pt(IV)Ac-POA. Magnification 60X, bar of 25 µm


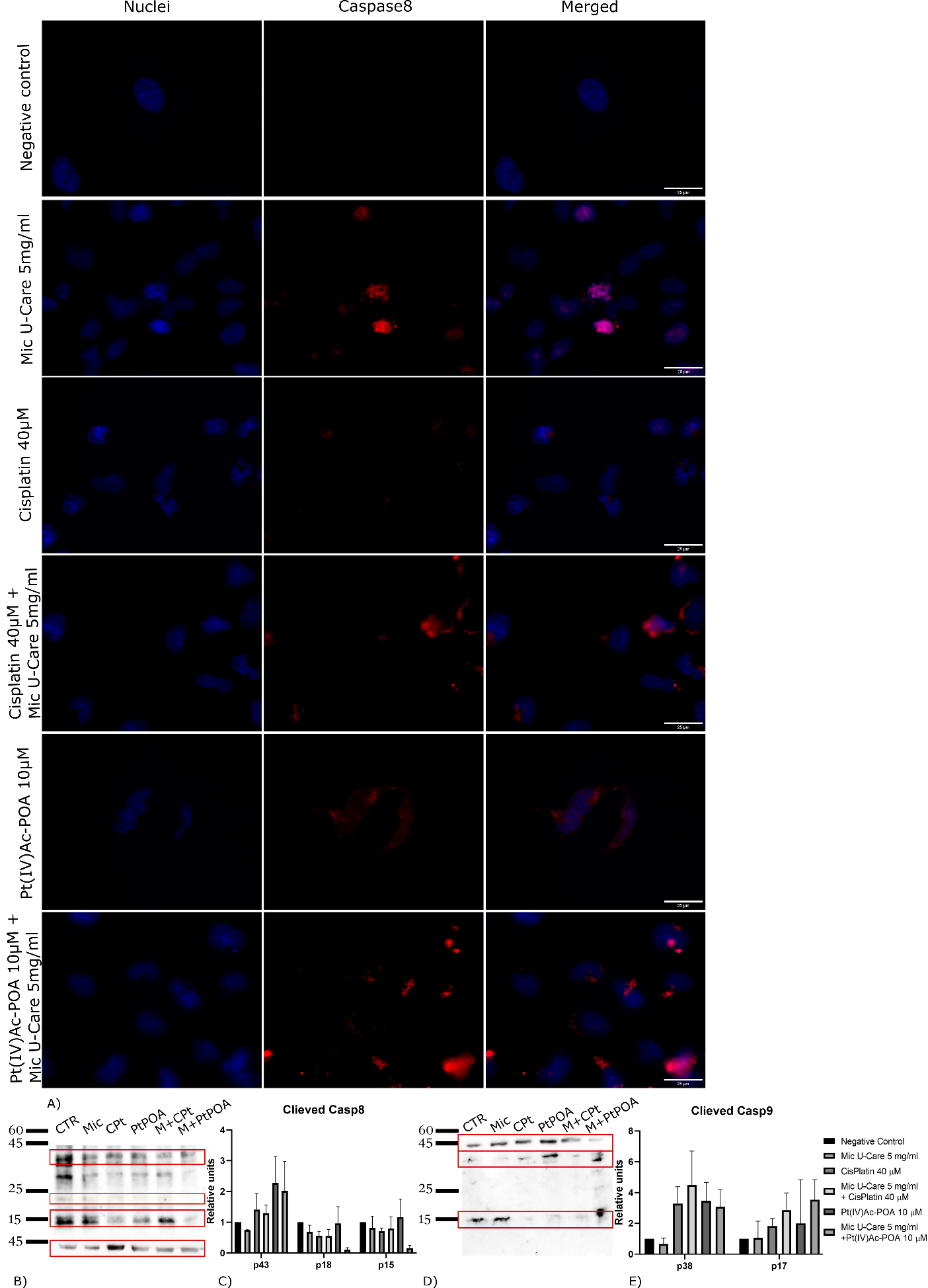


**Supplementary material 2** A) Immunolabeling for Clieved Caspase 8 (in red), DNA was stained with Hoechst 33258 (blue fluorescence), in U251 in control condition, and after 48 h-CT with Micotherapy U-Care 5 mg/ml, with CDDP 40 μM, with Pt(IV)Ac-POA 10 μM, with Mic U-Care + CDDP, with Mic U-Care + Pt(IV)Ac-POA. Magnification 60X, bar of 25 µm B) Western blot membrane of Cleaved Caspase 8 (43kDa, 18kDa, 15kDa) and Beta Actin (43kDa) C) Bar-graph representing mean+SEM of associated quantification of the bands for p43, p18, p15 fragments of activated Caspase 8 D) Western blot membrane of Cleaved Caspase 9 (38kDa, 17kDa) and Beta Actin (43kDa) E) Bar-graph representing mean+SEM of associated quantification of the bands for p38, p17 fragments of activated Caspase 9


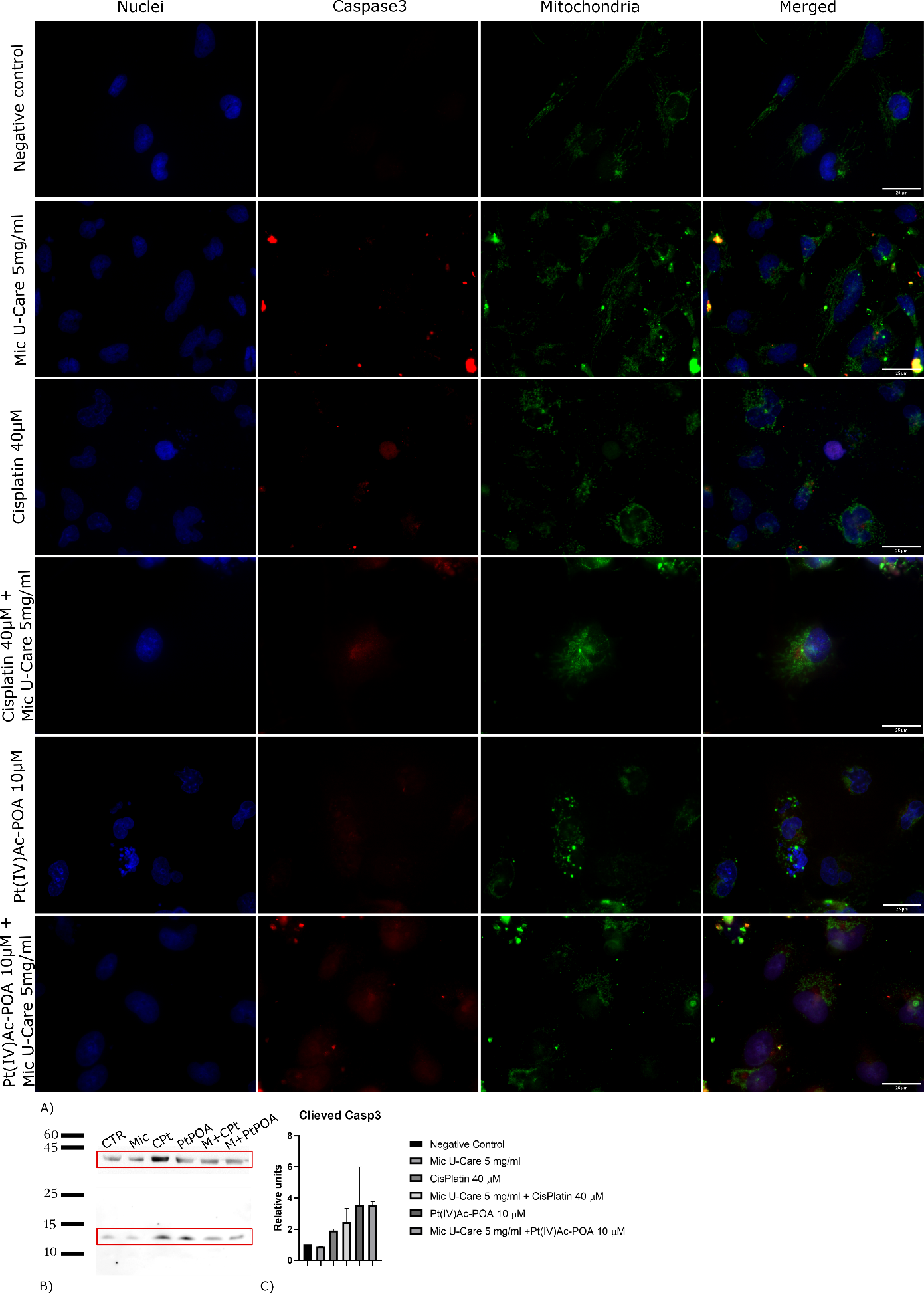


**Supplementary material 3** A) Immunolabeling for Clieved Caspase 3 (in green) and mitochondria (in red), DNA was stained with Hoechst 33258 (blue fluorescence), in U251 in control condition, and after 48 h-CT with Micotherapy U-Care 5 mg/ml, with CDDP 40 μM, with Pt(IV)Ac-POA 10 μM, with Mic U-Care + CDDP, with Mic U-Care + Pt(IV)Ac-POA. Magnification 60X, bar of 25 µm B) Western blot membrane of Cleaved Caspase 3 (12kDa) and Beta Actin (43kDa) C) Bar-graph representing mean+SEM of associated quantification of the bands for p12 fragments of activated Caspase 3


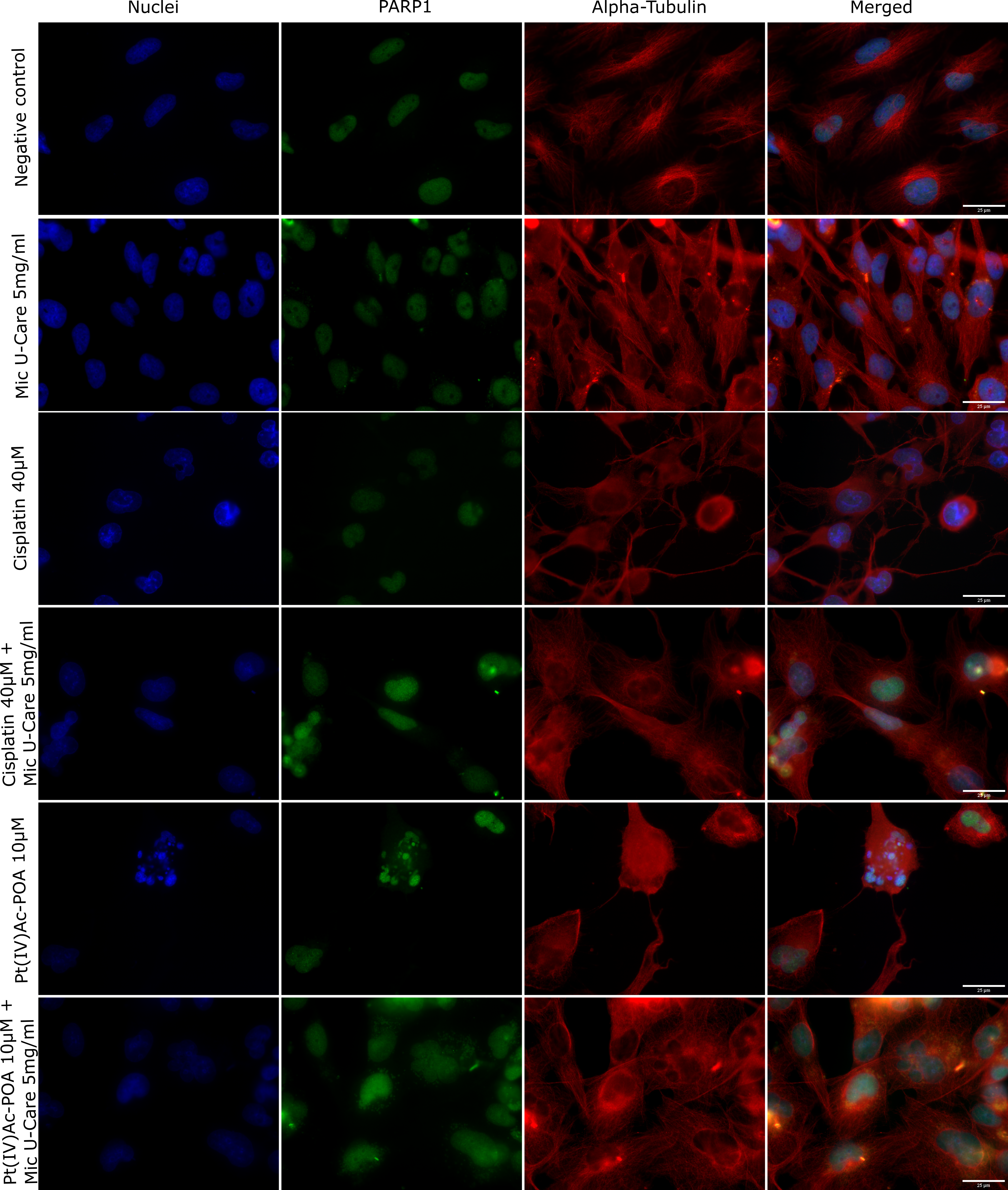


**Supplementary material 4** Immunolabeling for PARP1 (in green) and Alpha Tubulin (in red), DNA was stained with Hoechst 33258 (blue fluorescence), in U251 in control condition, and after 48 h-CT with Micotherapy U-Care 5 mg/ml, with CDDP 40 μM, with Pt(IV)Ac-POA 10 μM, with Mic U-Care + CDDP, with Mic U-Care + Pt(IV)Ac-POA. Magnification 60X, bar of 25 µm


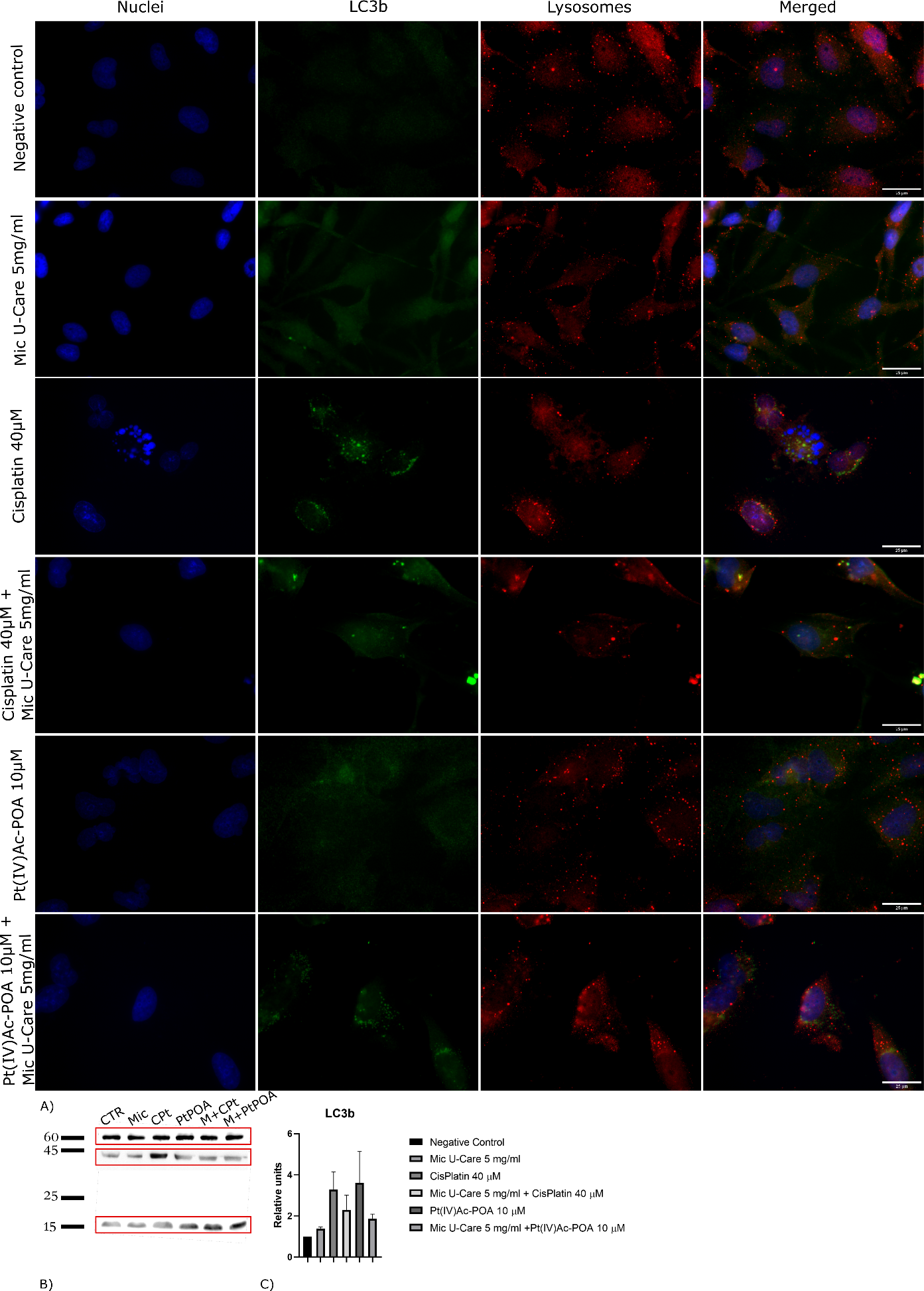


**Supplementary material 5** A) Immunolabeling for LC3b (in green) and lysosomes (in red), DNA was stained with Hoechst 33258 (blue fluorescence), in U251 in control condition, and after 48 h-CT with Micotherapy U-Care 5 mg/ml, with CDDP 40 μM, with Pt(IV)Ac-POA 10 μM, with Mic U-Care + CDDP, with Mic U-Care + Pt(IV)Ac-POA. Magnification 60X, bar of 25 µm B) Western blot membrane of LC3b (16kDa), SQSTM1/p62 (62kDa) and Beta Actin (43kDa) C) Bar-graph representing mean+SEM of associated quantification of the bands for p16 fragments of LC3bII


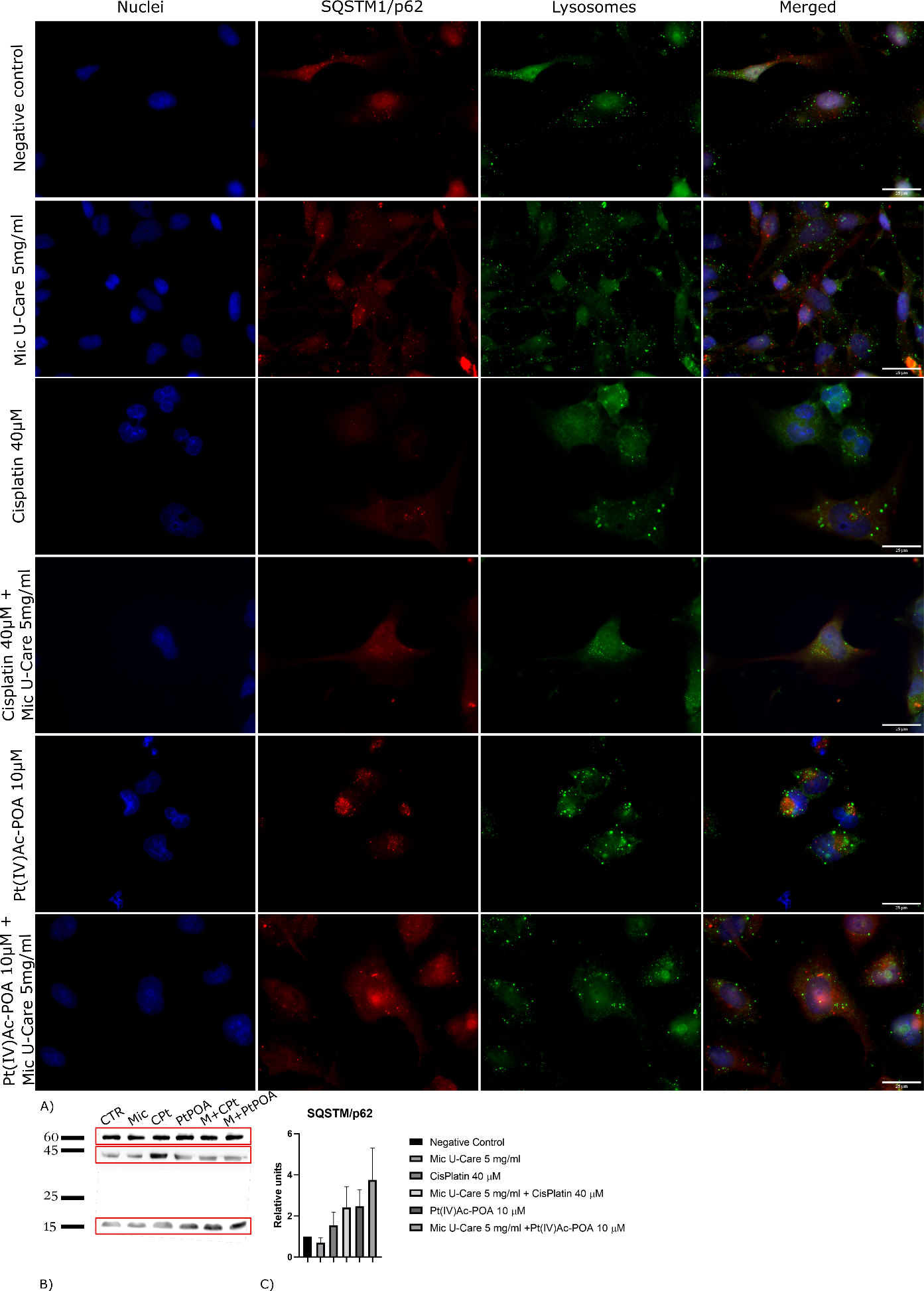


**Supplementary material 6** A) Immunolabeling for SQSTM1/p62 (in green) and lysosomes (in red), DNA was stained with Hoechst 33258 (blue fluorescence), in U251 in control condition, and after 48 h-CT with Micotherapy U-Care 5 mg/ml, with CDDP 40 μM, with Pt(IV)Ac-POA 10 μM, with Mic U-Care + CDDP, with Mic U-Care + Pt(IV)Ac-POA. Magnification 60X, bar of 25 µm B) Western blot membrane of LC3b (16kDa), SQSTM1/p62 (62kDa) and Beta Actin (43kDa) C) Bar-graph representing mean+SEM of associated quantification of the bands for p62 fragments of SQSTM1/p62


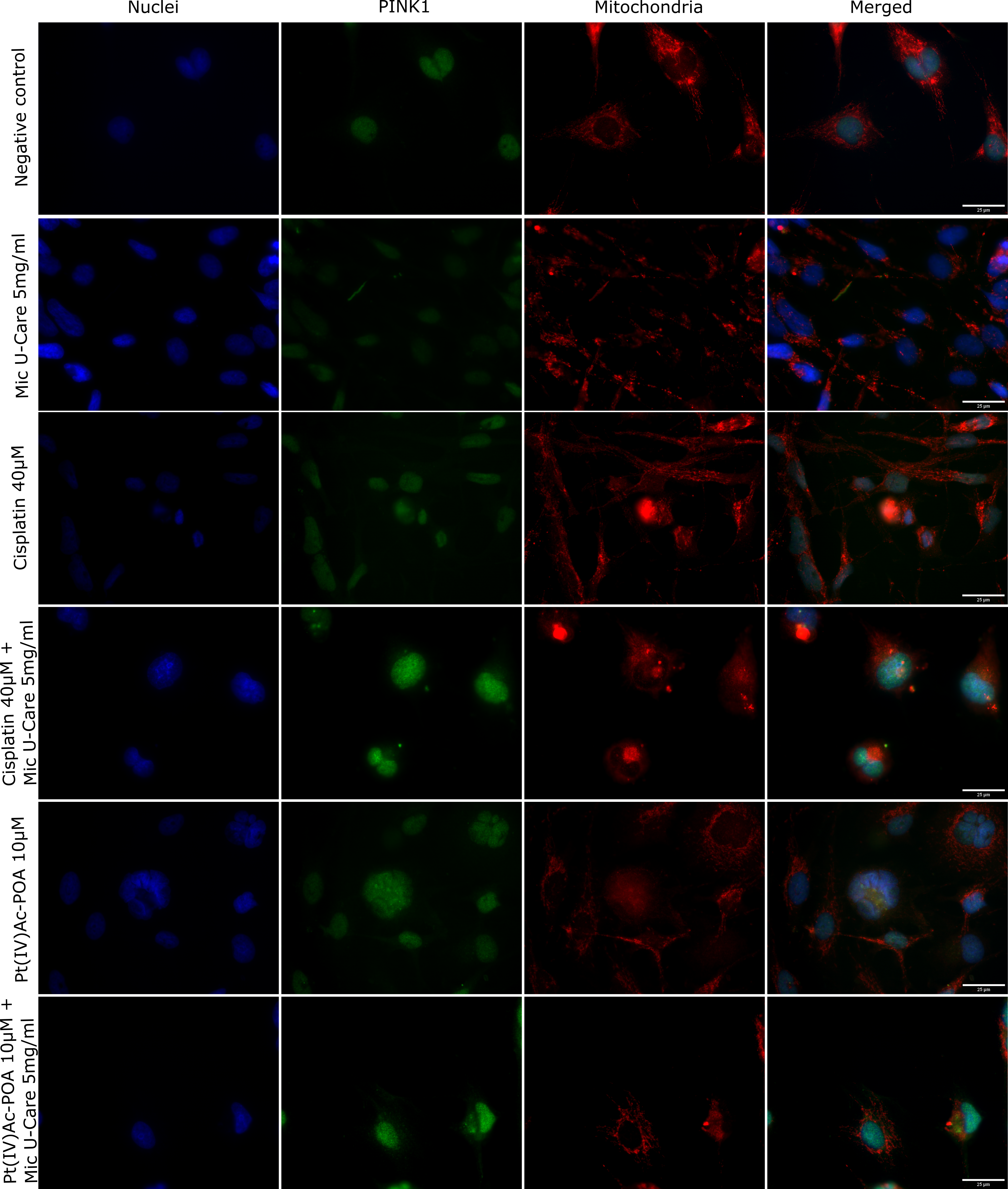


**Supplementary material 7** Immunolabeling for PINK1 (in green) and mitochondria (in red), DNA was stained with Hoechst 33258 (blue fluorescence), in U251 in control condition, and after 48 h-CT with Micotherapy U-Care 5 mg/ml, with CDDP 40 μM, with Pt(IV)Ac-POA 10 μM, with Mic U-Care + CDDP, with Mic U-Care + Pt(IV)Ac-POA. Magnification 60X, bar of 25 µm


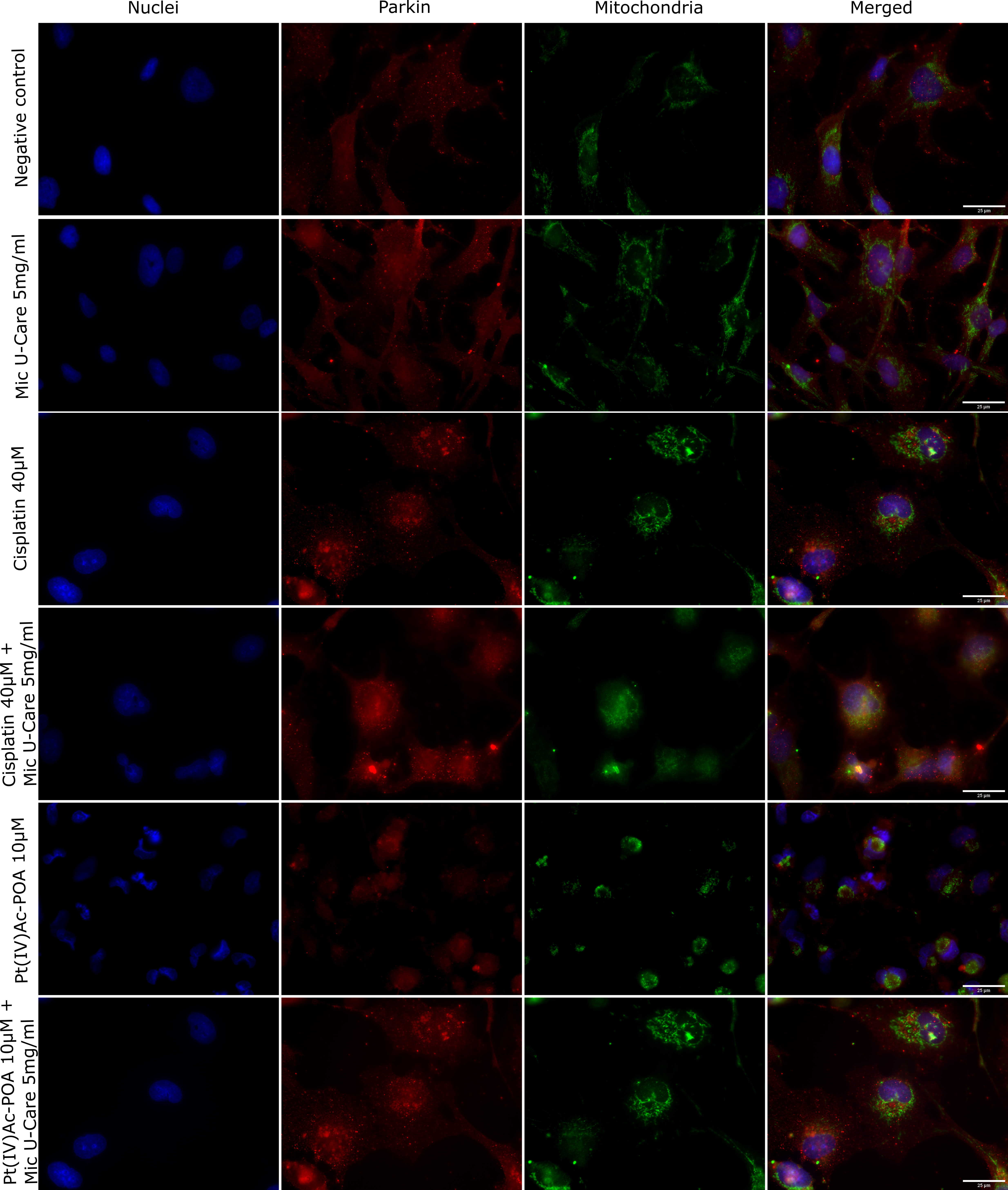


**Supplementary material 8** Immunolabeling for Parkin (in red) and mitochondria (in green), DNA was stained with Hoechst 33258 (blue fluorescence), in U251 in control condition, and after 48 h-CT with Micotherapy U-Care 5 mg/ml, with CDDP 40 μM, with Pt(IV)Ac-POA 10 μM, with Mic U-Care + CDDP, with Mic U-Care + Pt(IV)Ac-POA. Magnification 60X, bar of 25 µm


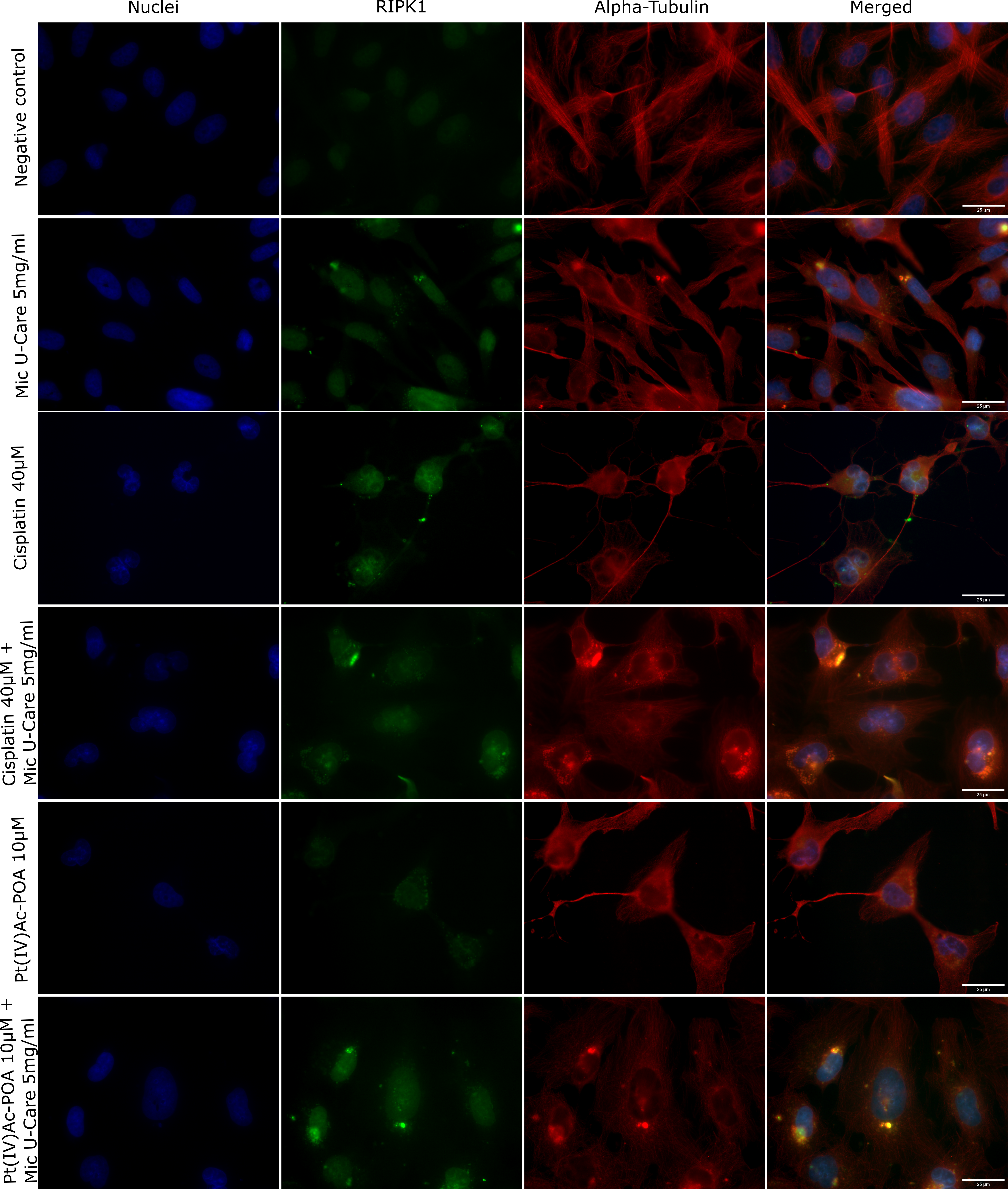


**Supplementary material 9** Immunolabeling for RIP1 (in green) and Alpha tubulin (in red), DNA was stained with Hoechst 33258 (blue fluorescence), in U251 in control condition, and after 48 h-CT with Micotherapy U-Care 5 mg/ml, with CDDP 40 μM, with Pt(IV)Ac-POA 10 μM, with Mic U-Care + CDDP, with Mic U-Care + Pt(IV)Ac-POA. Magnification 60X, bar of 25 µm


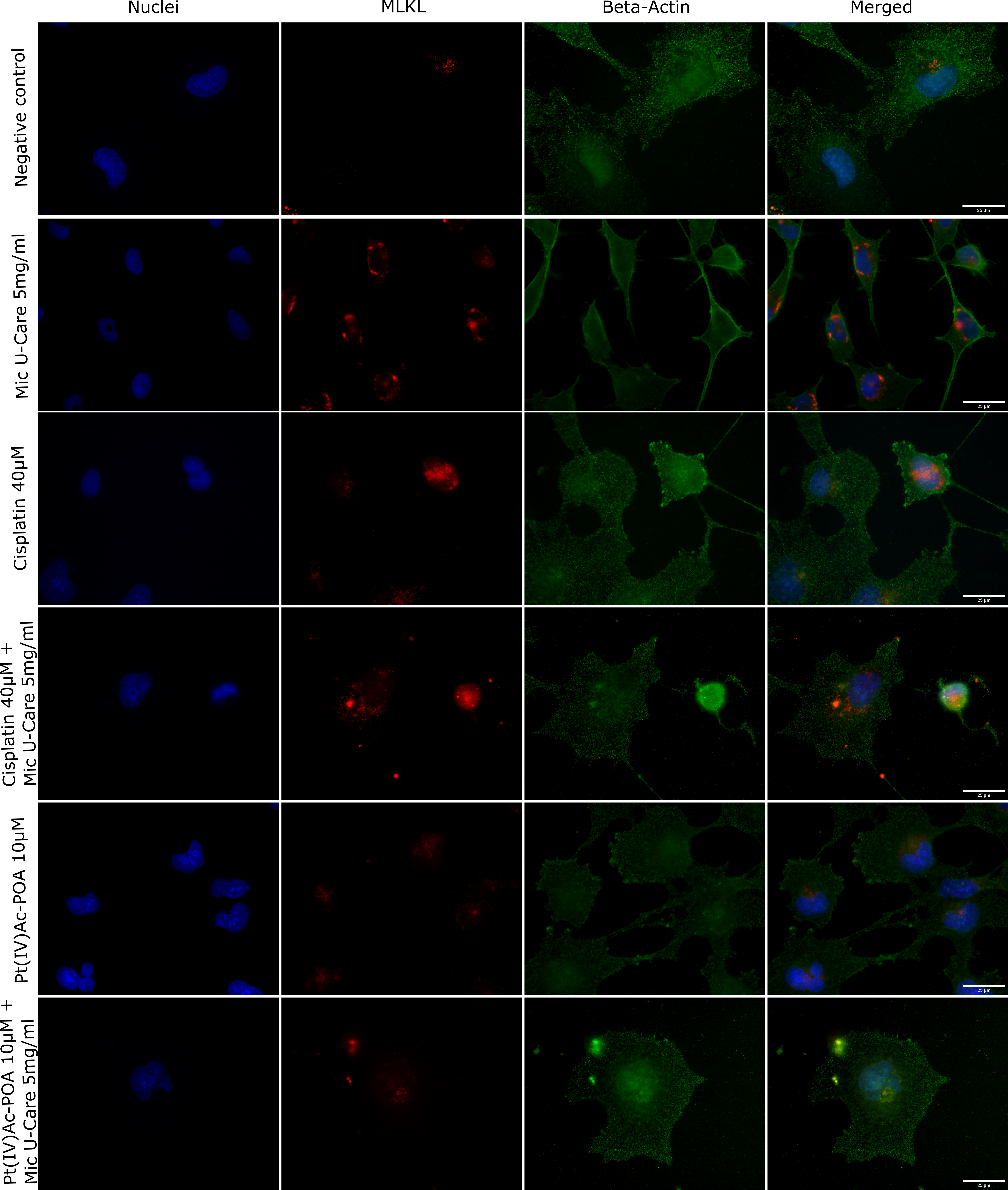


**Supplementary material 10** Immunolabeling for MLKL (in red) and Beta Actin (in green), DNA was stained with Hoechst 33258 (blue fluorescence), in U251 in control condition, and after 48 h-CT with Micotherapy U-Care 5 mg/ml, with CDDP 40 μM, with Pt(IV)Ac-POA 10 μM, with Mic U-Care + CDDP, with Mic U-Care + Pt(IV)Ac-POA. Magnification 60X, bar of 25 µm
